# Cerebral organoids transplantation repairs infarcted cortex and restores impaired function after stroke

**DOI:** 10.1038/s41536-023-00301-7

**Published:** 2023-05-30

**Authors:** Shi-Ying Cao, Di Yang, Zhen-Quan Huang, Yu-Hui Lin, Hai-Yin Wu, Lei Chang, Chun-Xia Luo, Yun Xu, Yan Liu, Dong-Ya Zhu

**Affiliations:** 1 Department of Pharmacology, School of Pharmacy, Nanjing Medical University, Nanjing, 211166 China; 2 Department of Neurology, Affiliated Drum Tower Hospital of Nanjing University, Nanjing, 210008 China; 3 Institution of Stem Cells and Neuroregeneration, Nanjing Medical University, Nanjing, 211166 China

**Keywords:** Regeneration and repair in the nervous system, Stroke

## Abstract

Stroke usually causes prolonged or lifelong disability, owing to the permanent loss of infarcted tissue. Although a variety of stem cell transplantation has been explored to improve neuronal defect behavior by enhancing neuroplasticity, it remains unknown whether the infarcted tissue can be reconstructed. We here cultured human cerebral organoids derived from human pluripotent stem cells (hPSCs) and transplanted them into the junction of the infarct core and the peri-infarct zone of NOD-SCID mice subjected to stroke. Months later, we found that the grafted organoids survived well in the infarcted core, differentiated into target neurons, repaired infarcted tissue, sent axons to distant brain targets, and integrated into the host neural circuit and thereby eliminated sensorimotor defect behaviors of stroke mice, whereas transplantation of dissociated single cells from organoids failed to repair the infarcted tissue. Our study offers a new strategy for reconstructing infarcted tissue via organoids transplantation thereby reversing stroke-induced disability.

## Introduction

Stroke is one of the leading causes of death and adult disability worldwide^1,2^. The disability after stroke is usually caused by the permanent loss of infarcted tissue. Recently, clinical treatments have improved in the acute time window, such as intravenous thrombolysis and endovascular thrombectomy to rescue the penumbra area after ischemia^3,4^, but therapeutics during repair phase is still limited to physical rehabilitation, disability remains one of the key challenges to recovery from stroke^1,5,6^.

Stroke-induced neuronal loss is a primary cause of motional and cognitive functional disorders for patients^7^. Stem cell-based therapies aiming to promote neurogenesis and replacement of lost neurons offer promise because of their potential to provide neurorestorative benefits to stroke^8,9^. However, whether endogenous neurogenesis exists in the adult human brain has been controversial, recent studies indicate that few putative new neurons exist in the adult human hippocampus^10,11^. Various types of stem cells or neural progenitors have been used in stroke animals^12,13^. Clinical trials also have been using intravenous delivery of human bone marrow- and cord blood-derived mesenchymal stem cells^14,15^, or using intracerebral delivery of pluripotent fetal progenitors^16–18^ and human embryonic stem cells (hESCs)-derived neural progenitors^19^. However, the therapeutic effects of cell therapy are limited, because it is difficult to repair the tissue structure in the infarcted core.

Recently, scientists have been able to make advances in stem cell-derived 3D human brain organoids^20,21^, using human pluripotent stem cells (hPSCs) combined with biomaterials and genome techniques. A variety of protocols for preparing human brain organoids have been published^22^, the main purpose of which is to study early neural development and the pathogenesis of neuropsychiatric disorders^23–26^. Methods for transplanting human brain organoids into the adult mouse brain have been established and organoid grafts showed neuronal differentiation, axonal projection, and integration into host neuronal circuits in the brain in normal mice and newborn athymic rats, representing important research progress in the field of brain organoids integration into neuronal circuits of the host brain^27,28^. However, there is still a lack of solid evidence on whether human brain organoids transplantation after stroke can repair the infarcted tissue, thereby reversing stroke-induced disability. Here, we cultured human cerebral organoids in vitro through the directional differentiation of hPSCs and transplanted them into the junction of the infarct core and the peri-infarct zone of the non-obese diabetes-severe combined immunodeficiency (NOD-SCID) mice subjected to photothrombotic stroke. We found that the cerebral organoids survived well in the infarcted core, differentiated into target neurons, sent axons to distant brain targets, including callosal, corticostriatal, corticothalamic, and corticospinal projections, and integrated into the host neural circuit. More importantly, the transplantation of human cerebral organoids repaired the infarcted tissue structure and substantially eliminated sensorimotor defect behaviors of stroke mice.

## Results

### The grafted cerebral organoids recapitulate characteristics of the cerebral cortex in stroke mice

Following our previously established protocols in vitro^29,30^, hPSCs were differentiated into human cerebral organoids (Supplementary Fig. 1a, b). On day 23 after cerebral organoids differentiation, rosette-like structures could be observed. Organoids contained the ventricular-like zone (VZ), expressing dorsal forebrain progenitor marker paired box protein Pax-6 (PAX6), neural stem cells marker Nestin, VZ marker SOX2 (also known as sex-determining region Y-box 2), intermediate progenitor marker T-box brain protein 2 (TBR2), the apical marker protein kinase C-λ (PKC-λ), immature neurons marker doublecortin (DCX), outer radial glial cells marker HOPX and neural cell adhesion molecule marker NCAM (Supplementary Fig. 1c–f). On day 40 after differentiation, the basal surface cells of cerebral organoids gradually matured, expressing mature neuron marker NeuN, while the apical surface cells were still in proliferation and expressed Ki67 (Supplementary Fig. 1g). At the same time, organoids expressed forebrain marker forkhead box protein G1 (FOXG1) and neuron-specific class III beta-tubulin (Tuj-1) (Supplementary Fig. 1h). After continuous culture for 50–100 days, organoids formed cortical plates. On day 100 after differentiation, cerebral organoids expressed layer V/VI markers COUP-TF-interacting protein 2 (CTIP2), forkhead box protein P2 (FOXP2), T-box brain protein 1 (TBR1) and layer II-IV markers special AT-rich sequence-binding protein 2 (SATB2) and BRN2 (POU domain, class 3, transcription factor 2) (Supplementary Fig. 1i–k), showing a preliminary inside-out neurogenesis pattern. On day 190 after differentiation, many neurons in cerebral organoids were excitatory glutaminergic neurons expressing glutamate and mature neuronal marker microtubule-associated protein 2 (MAP2) and a few cells expressed inhibitory GABAergic neuronal marker gamma-aminobutyric acid (GABA) and astrocytes marker glial fibrillary acidic protein (GFAP) (Supplementary Fig. 1l–n), indicating that cerebral organoids mimic the early stage of human cortex patterning in vitro.

**Fig. 1:**
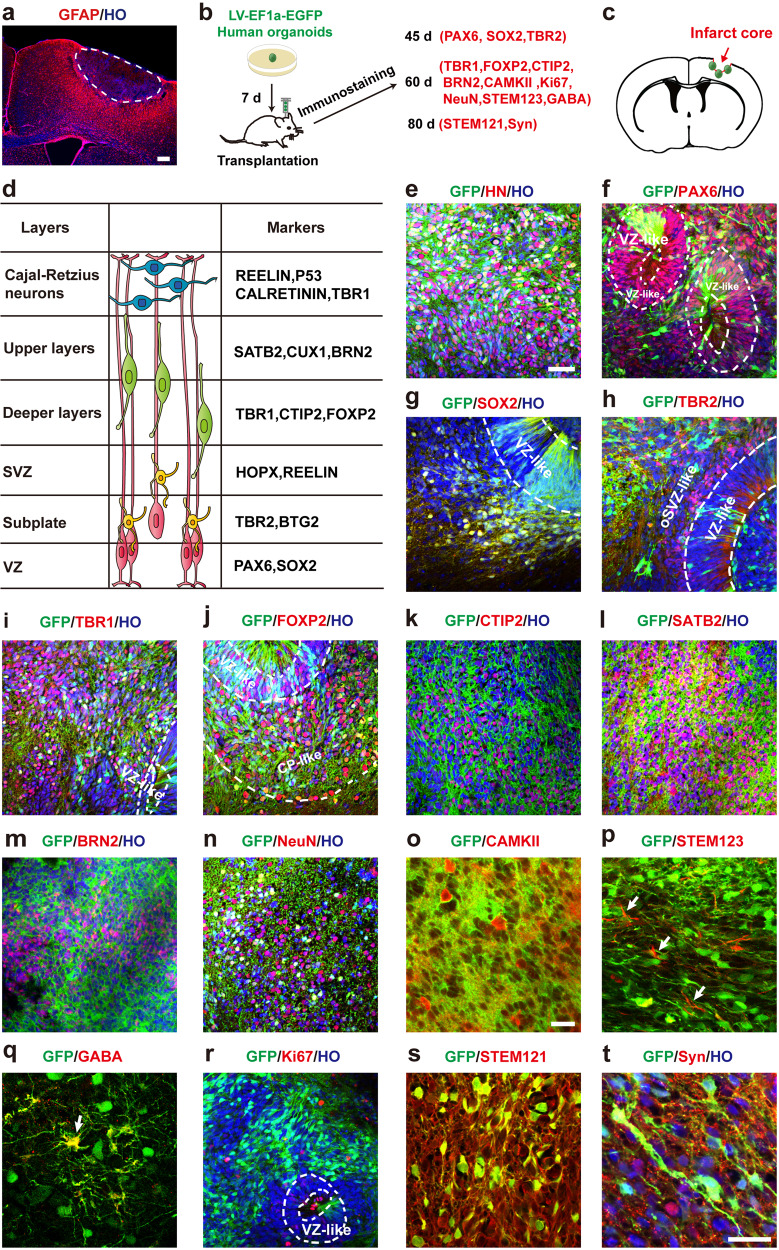
Grafted human cerebral organoids recapitulate characteristics of the cerebral cortex in stroke mice. **a** Representative image showing the cerebral cortex of mice stained with GFAP on d 3 after stroke. The area outlined by the white dotted line indicates the infarct core. Scale bar, 200 μm. **b** Diagram showing human cerebral organoids transduced with LV-EF1a-EGFP and their transplantation, timeline of immunostaining. **c** Diagram showing the site of the organoids transplant. **d** Schematic showing corticogenesis of developing human cerebral cortex. **e**–**h** Images showing GFP^+^ grafts expressing VZ markers PAX6 and SOX2 and subplate marker TBR2 at 45 d after transplantation. **i**–**m** Images showing GFP^+^ grafts expressing deeper layers markers TBR1, FOXP2 and CTIP2 and upper layers markers SATB2 and BRN2 at 60 d after transplantation. Images showing GFP^+^ grafts expressing NeuN (**n**), CaMKII (**o**), STEM123 (**p**) GABA (**q**) and Ki67 (**r**) at 60 d after transplantation. Images showing GFP^+^ grafts expressing STEM121 (**s**) Syn (**t**) at 80 d after transplantation. Scale bars, **e**–**n** and **r**, 50 μm, **o**–**q** and **s**, 20 μm, **t**, 20 μm. HO Hoechst 33258 dye.

To determine whether human cerebral organoids recapitulate characteristics of the cerebral cortex after transplantation into stroke brain, we induced photothrombotic stroke in the forelimb motor cortex (PT-1) or parietal cortex (PT-2) of NOD-SCID mice independently. No significant difference in infarct volume was observed between PT-1 mice and PT-2 mice 3 days after the stroke (Supplementary Fig. 2a, b). However, PT-1 mice displayed a significantly increased number of left-foot faults in the grid-walking test and asymmetry index in the cylinder test, and significantly increased touch latency and removal latency in the adhesive-removal test (Supplementary Fig. 2c–f), while PT2 mice showed slightly increased asymmetry index in the cylinder test (Supplementary Fig. 2d). This slight change may be caused by visual impairment which was partially damaged in the PT2 mice. Therefore, a small infarct in the forelimb motor cortex can cause marked motor behavioral deficits. Moreover, because a large number of activated astrocytes (GFAP^+^) were arranged radially around the infarcted core on day 3 after stroke, the infarct core and peri-infarct area could be clearly distinguished (Fig. 1a).

Before transplantation, we analyzed the cell composition of organoids derived from hESCs, which differentiated for 50 days in vitro. As shown in Supplementary Fig. 3a–h, the ratio of all cells expressed FOXG1, Tuj-1, PAX6, TBR1, CTIP2, glutamic acid decarboxylase 67(GAD67), Ki67 and NeuN was 95.7%, 94.9%, 25.6%, 11.2%, 12.4%, 4.3%, 9.1%, and 11.1% respectively (Supplementary Fig. 3i), showing features of forebrain precursor cells. We also prepared the cerebral organoids from IMR-90–4 iPSCs and observed similar results (Supplementary Fig. 3j–n). On day 7 after stroke, human cerebral organoids transduced with lentivirus LV-EF1a-EGFP one week ahead were transplanted into the junction of the infarct core and the peri-infarct zone of mice, on the left, right, and bottom of the infarct core, respectively (Fig. 1b, c and Supplementary Fig. 4b). Immunostaining showed that transplantation of three organoids instead of one could completely fill the infarcted cavity on day 60 after transplantation (Supplementary Fig. 4). Therefore, in the follow-up experiment, we transplanted three organoids.

Mimicking the corticogenesis of developing human cerebral cortex, grafted human cerebral organoids (GFP^+^) expressed VZ markers PAX6 and SOX2 and subplate marker TBR2, forming a rosette-like VZ structure on day 45 after transplantation (Fig. 1d–h). We compared the organoids cultured in vitro for 95 days with the organoids cultured in vitro for 50 days and then transplanted into brain for 45 days and found that the transplantation significantly increased expressions of the progenitor markers PAX6 and SOX2 (Supplementary Fig. 5a–c), suggesting that the maturation of organoids slows down after transplantation into the brain. Moreover, the grafted organoids expressed deeper layers markers TBR1, FOXP2 and CTIP2, and upper layers markers SATB2 and BRN2, and expressed NeuN and pyramidal neurons marker Calcium/calmodulin-dependent protein kinase II (CaMKII) on day 60 after transplantation (Fig. 1i–o); and meanwhile, a small number of GFP^+^ cells expressed human astrocyte marker STEM123 and GABA, and a few GFP^+^ cells expressed cell proliferation marker Ki67 (Fig. 1p–r). On day 80 after transplantation, GFP^+^ cells were stained by human neurite marker STEM121 and graft-derived fibers expressed synaptophysin (Syn) (Fig. 1s, t). We quantitatively analyzed the proportion of the above markers expressed in the graft cells (labeled with human nuclei (HN) (Supplementary Fig. 6b–j, p) or GFP (Supplementary Fig. 6k–o, q), suggesting that the grafted organoids in the infarcted cortex of stroke mice can survive for a long time and differentiate into cortical neurons in a time-dependent manner.

### Repair of infarcted tissue via transplanting human cerebral organoids

To further test the long-term differentiation of grafted human cerebral organoids in the cortex of stroke mice, we detected the cellular composition of cerebral organoids in the cortex of stroke mice on day 180 after transplantation. As shown in Supplementary Fig. 7, the proportion of HN^+^ cells expressing TBR1, CTIP2 and SATB2 was 19.0%, 42.9% and 44.1% (Supplementary Fig. 7a–c, j), respectively, and the proportion of HN^+^ cells expressing NeuN, DCX (an immature neuron marker), Ki67, GFAP, oligodendrocyte transcription factor 2 (OLIG2) and GABA was 44.5%, 43.9%, 0.6%, 6.2%, 4.6% and 2.1% respectively (Supplementary Fig. 7d–I, j). We analyzed the source of OLIG2 in the grafts and found that OLIG2^+^/HN^+^ and OLIG2^+^/HN^−^ cells were 92.7% and 7.3% respectively (Supplementary Fig. 7k–l), revealing that oligodendrocytes are mainly derived from grafted human cerebral organoids. These data suggest that grafted human cerebral organoids are mainly composed of mature and immature glutaminergic neurons in the infarcted cortex, with a small number of astrocytes, oligodendrocytes and GABAergic neurons 180 d after transplantation.

**Fig. 2:**
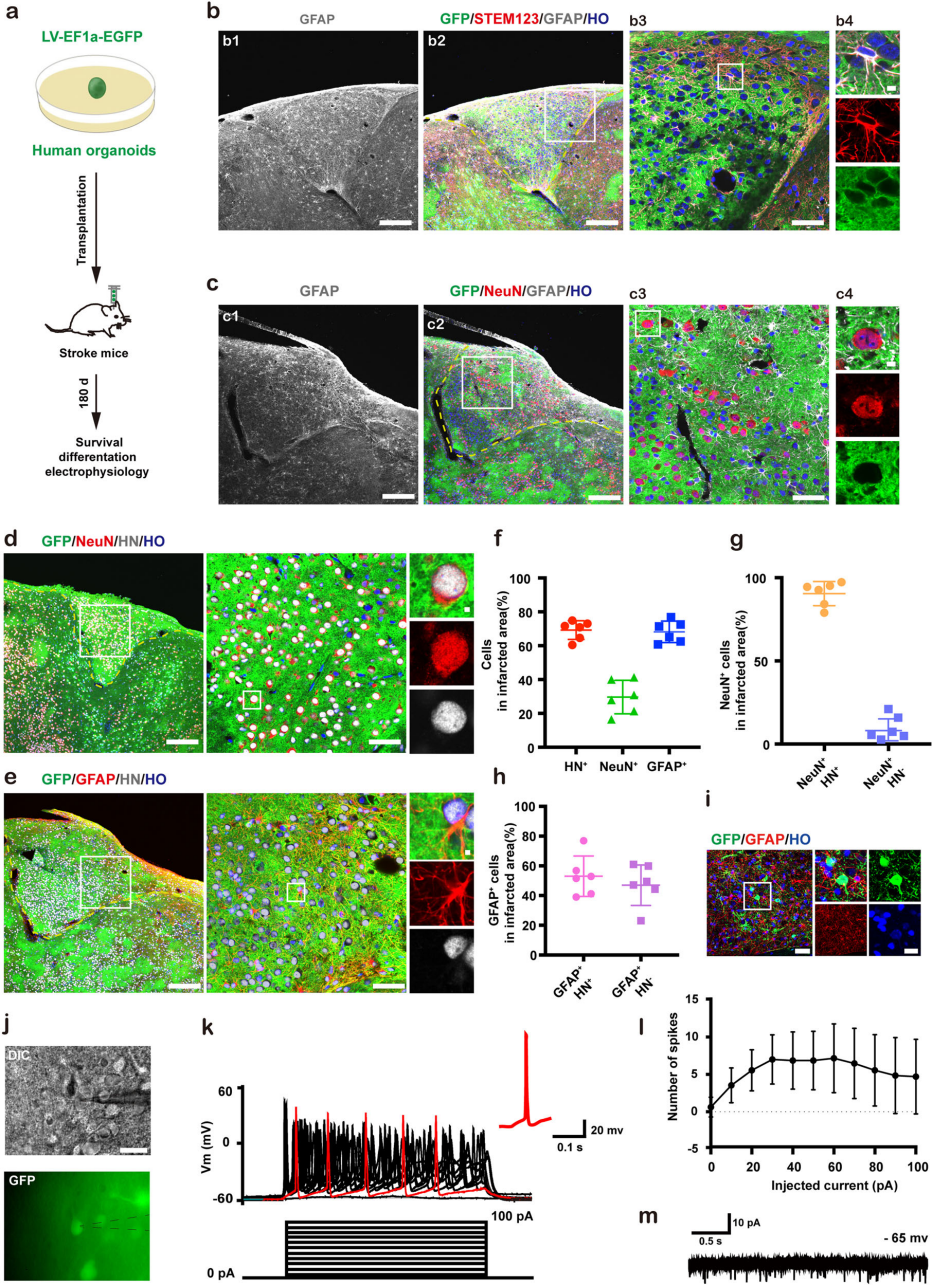
Survival and differentiation and functions of grafted human cerebral organoids in the infarct core of stroke mice. **a** Design of the experiments for **b**–**m**. Representative images showing the coronal section stained with STEM123 and GFAP in animal A (**b**) or with NeuN and GFAP in animal B (**c**) on d 180 after organoids transplantation, in which, **b1** and **c1** showing infarct area (scale bar, 200 μm), **b2** and **c2** showing the survival of organoids (scale bar, 200 μm), the boxed area in **b2** and **c2** was shown in high magnification in **b3** and **c3** respectively (scale bar, 50 μm), the boxed area in **b3** and **c3** was shown in high magnification in **b4** and **c4** respectively (scale bar, 5 μm). Representative images showing HN^+^ graft cells co-labeled with NeuN (**d**) or GFAP (**e**) (left, scale bar, 200 μm) on d 180 after transplantation. The boxed area in leftward image was shown in high magnification on the middle image (scale bar, 50 μm), and the boxed area in middle image was shown in high magnification on the right three panels (scale bar, 5 μm). **f**–**h** Bar graphs show cerebral organoids’ cellular composition at 180 d after transplantation. *n* = 6 animals. More than 4,500 cells from random fields were manually counted in each condition. **i** Representative image showing organoids neurons (GFP^+^) and astrocytes (GFAP^+^) (upper, scale bar, 50 μm) and high magnification images from a selected area in the upper image (lower, scale bar, 20 μm) at 180 d after transplantation. **j** Images showing whole-cell patch-clamp recordings from organoids neurons (GFP^+^). Black dotted lines indicate electrode. Scale bar, 40 μm. **k** Voltage traces showing APs from GFP^+^ neuron in response to current-step, in which, red traces showing the first AP induced by 0 pA injected current. **l** Number of APs evoked by various current steps from GFP^+^ neurons. *n* = 13 neurons from 5 mice. **m** Representative traces of sPSCs from the organoids neurons. *n* = 34 neurons from 7 mice. In **b**–**e**, the area outlined by the yellow dotted line indicates the infarct core. In **f**–**h** and **l**, data were presented as mean ± standard deviation. HO Hoechst 33258 dye, HN Human Nuclei.

Cell therapy with single neural stem cell is difficult to repair the tissue structure in the infarcted core^31,32^. To test whether the grafted human cerebral organoids can repair infarcted tissue, we transplanted the human cerebral organoids transduced with LV-EF1a-EGFP into the junction of the infarct core and the peri-infarct zone of stroke mice on day 7 after stroke and assessed the long-term survival and differentiation of grafts in the infarct core on day 180 after transplantation (Fig. 2a). We observed that the infarcted core was fully filled by grafted organoids, with astrocytes labeled by STEM123^+^ (specifically with GFAP of human cells marker) (Fig. 2b) and mature neurons labeled by NeuN (Fig. 2c), suggesting long-term survival of organoids in the infarcted core. To determine whether the transplantation of dissociated single cells from organoids can repair infarcted tissue-like organoids transplantation, we prepared the dissociated single cells from equal numbers of organoids and transplanted them into the junction area as above. Although the total number of cells was comparable and the location of transplants was the same between the single cells and organoids transplantation, only a few cells survived at the infarct margin on day 180 after the dissociated organoids cells transplantation, and in the peri-infarct area, although there were many GFP^+^ fibers, few GFP^+^ neurons were observed (Supplementary Fig. 8). Thus, organoids but not dissociated organoids cells transplantation can repair the infarcted tissue after stroke.

To assess the long-term differentiation of human cerebral organoids in the infarcted core, immunofluorescence staining was performed. As shown in Fig. 2d, e, a large number of HN^+^ organoids cells were co-labeled by GFAP or NeuN. Approximately 70% of the cells in the infarcted core were HN-positive, and the percentage of HN^+^ cells expressing GAFP and NeuN was 65.7% and 29.6% respectively (Fig. 2f), suggesting that the cerebral organoids in the infarcted core are more likely to differentiate into glial cells, different from the neuronal differentiation of the grafts in the peri-infarct area. The source analysis of GFAP- and NeuN-positive cells in the infarcted core showed that GFAP^+^/HN^+^ and GFAP^+^/HN^−^ cells were 53.1% and 46.9% respectively, and NeuN^+^/HN^+^ and NeuN^+^/HN^−^ cells was 90.4% and 9.6% respectively (Fig. 2g, h), indicating that more than half of astrocytes are derived from the host, while neurons are predominantly derived from grafted human cerebral organoids. However, in the neural induction medium (NIM)-treated stroke mice, the infarcted core had become a cavity with few cells surviving on day 180 after NIM microinjection (Supplementary Fig. 9).

To test whether the organoids-derived neurons in the infarcted core have a normal physiological function, we detected their electrophysiological characteristics on day 180 after transplantation (Fig. 2a, 2i–m). The electrophysiological recordings showed that the EGFP^+^ organoids neurons in the infarcted core generated action potentials (APs) in response to membrane depolarization induced by current-step (Fig. 2i–l) and spontaneous postsynaptic currents (sPSCs) (Fig. 2m), suggesting that grafted cerebral organoids neurons exhibit electrophysiological properties of mature neurons.

### Integration of grafted cerebral organoids into the host neural circuits

**Fig. 3:**
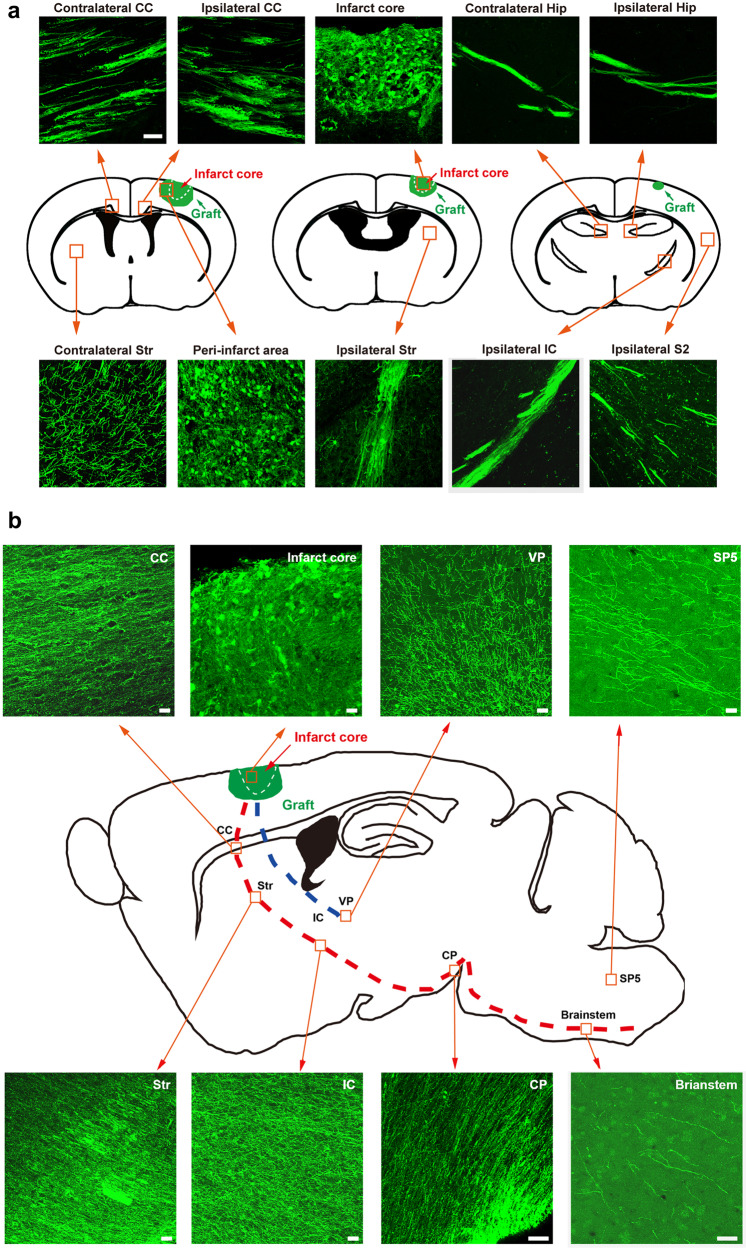
Grafted cerebral organoids extend long-distance projections to specific brain areas in stroke mice 180 days after transplantation. **a** Schematic showing the GFP^+^ grafts in the infarct core and peri-infarct area on the coronal sections of brain (middle), and images showing graft-derived projections in the contralateral and ipsilateral corpus callosum (CC), hippocampus (Hip) (upper) and striatum (Str), ipsilateral S2 and internal capsule (IP). Scale bar, 50 μm. **b** Schematic showing the GFP^+^ grafts in the infarct core and peri-infarct area on the sagittal sections of brain (middle), and images showing graft-derived projections in the CC, and striatum, IC, cerebral peduncle (CP), ventral posterior thalamic nucleus (VP), spinal trigeminal nucleus (Sp5) and brainstem. Scale bar, 20 μm.

The mammalian neocortex is responsible for processing multiple modalities of sensory information and controlling motor output. Glutamatergic projection neurons in the cortex send axons to distant brain targets^33^, in which, callosal projection neurons primarily located in layers II/III send axons to the contralateral cortex, intrahemispheric projection neurons located in layer IV send axons within a single cortical, corticostriatal projection neuron located in layer V send axons to contralateral and ipsilateral striatum, corticospinal motor neurons located in layer V send axons to spinal cord and corticothalamic projection neurons located in layer VI send axons to thalamus^34^. On day 180 after transplantation, massive of GFP^+^ nerve fibers appeared in the contralateral and ipsilateral corpus callosum (CC), striatum and hippocampus, ipsilateral internal capsule (IC), sensory cortex (S2) and ventral posterior thalamic nucleus (VP), suggesting that the grafted human cerebral organoids can establish commissural, associative and corticofugal projections in host brain, similar to the neocortex of mammals (Fig. 3a). More interestingly, the GFP^+^ axon bundles from the grafted human cerebral organoid passed through the CC to the striatum, and then passed through the IC and the cerebral peduncle (CP) to the brainstem (Fig. 3b), suggesting the establishment of the corticospinal tract (CST), a crucial circuit for the recovery of motor function from stroke. In addition, GFP^+^ fibers were also observed in the spinal trigeminal nucleus (Sp5), a somatosensory nucleus.

**Fig. 4:**
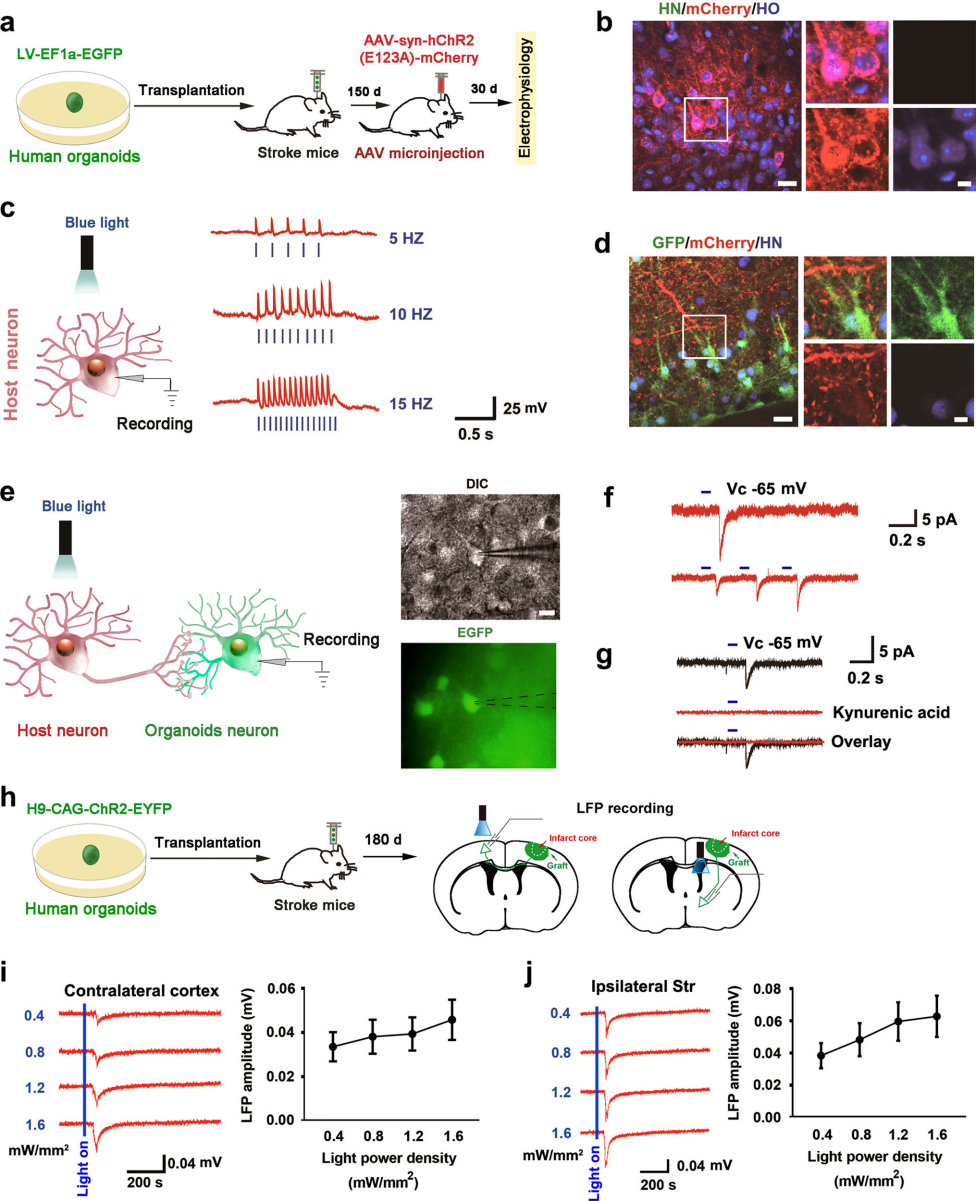
The grafted organoid neurons receive synaptic input from host cells and synaptic efferent from the organoid neurons to distant host neurons. **a** Design of the experiments for **b**–**g**. **b** A representative image showing that mCherry^+^ cells were not co-labeled with HN (left, scale bar, 20 μm) and high magnification images from a selected area in the leftward image (right, scale bar, 5 μm). **c** Diagram (left) showing the strategy to evoke APs in the host neuron and APs traces evoked by blue photo stimuli pulses (470 nm, 10 mW/mm^2^, 5 ms duration) of various frequencies on the ChR2-mCherry-expressing host neuron (right). **d** A representative image showing mCherry^+^ fibers extending to GFP^+^/HN^+^ human organoids neurons (left, scale bar, 20 μm) and high magnification images from a selected area in the leftward image (right, scale bar, 5 μm). **e** Diagram (left) showing the strategy to test whether organoids neurons receive synaptic input from host neurons and images (right) showing whole-cell patch-clamp recordings from grafted organoids neurons (GFP^+^). Black dotted lines indicate the placement of patch-clamp electrode. Scale bar, 20 μm. **f** Representative traces of PSCs evoked by 1 or 3 blue photo stimuli pulses at a holding potential −65 mV. **g** Representative traces of PSCs evoked by 1 blue photo stimuli pulse with or without kynurenic acid (α-amino-3-hydroxy-5-methyl-4-isoxazole propionate (AMPA) and N-methyl-D-aspartate (NMDA) receptors blocker). **h** Design of the experiments for **i** and **j**. **i**, **j** Cerebral organoids expressing ChR2 were grafted into the junction area between infarct core and peri-infarct zone of stroke mice and blue light-induced LFPs were recorded in the contralateral motor cortex and ipsilateral striatum. **i** Representative LFP traces recorded in the contralateral motor cortex (left, average of 20 responses) and input-output curves of peak amplitude for LFPs (right) (*n* = 7 slices from 3 animals). **j** Representative LFP traces recorded in the ipsilateral striatum (left, average of 20 responses) and input-output curves of peak amplitude for LFPs (right) (*n* = 6 slices from 3 animals). In **f** and **g**, *n* = 13 neurons from 5 mice, 6 recorded neurons were responsive to photostimulation. In **i** and **j**, data were presented as mean ± standard deviation. HO Hoechst 33258 dye, HN Human Nuclei.

To determine whether the neurons of grafted organoids receive the synaptic afferent from the host neuron and integrate into the host neural circuit, we microinjected an AAV-syn-hChR2(E123A)-mCherry into the adjacent area of the grafts on day 150 after transplantation, and 30 days later, we performed electrophysiological recordings (Fig. 4a). Immunostaining showed that less than 3% of mCherry^+^ cells were HN-positive (mCherry^+^/HN^+^) (Fig. 4b, Supplementary Fig. 10), indicating that almost all infected cells were host cells. Next, we illuminated the cortex in the acute slices with blue light (470 nm) and found that trains of short pulses of blue light-induced AP firing in ChR2-expressing neurons in current-clamp mode, and the AP firing probability followed the frequency of photostimulation, validating the functionality of expressed ChR2 (Fig. 4c). As shown in Fig. 4d, EFGP^+^ organoids neurons were surrounded by abundant mCherry^+^ fibers in the cortex, suggesting possible interactions between organoids neurons and host neurons. Next, we recorded postsynaptic currents (PSCs) in the GFP^+^ neurons by photostimulation of the mCherry^+^ fibers from the host neurons (Fig. 4e) and found that the illumination resulted in inward currents in GFP^+^ neurons under voltage-clamp recording conditions at a holding potential of −65 mV (Fig. 4f) and the PSCs were blocked by the application of ionotropic glutamate receptor antagonist kynurenic acid^35^ (Fig. 4g), suggesting a glutaminergic synaptic afferent from host neurons to the neurons of grafted organoids.

Next, we investigated whether there is synaptic efferent from the organoids neurons to host neurons. We generated human cerebral organoids from a previously reported hESC line expressing ChR2 fused with EYFP^29^, named H9-CAG-ChR2-EYFP cell line, and transplanted the organoids into the junction of the infarct core and the peri-infarct zone of stroke mice, and 180 days later, performed electrophysiological recordings (Supplementary Fig. 11a). Immunostaining showed that EYFP^+^ cells were HN-positive, indicating that they are grafted organoids-derived cells (Supplementary Fig. 11b). Trains of short pulses of blue light-induced AP firing in ChR2-expressing neurons in current-clamp mode, and the AP firing probability followed the frequency of photostimulation, validating the functionality of expressed ChR2 (Supplementary Fig. 11c). HN^−^ host neurons were surrounded by abundant GFP^+^ fibers in the cortex, suggesting possible interactions between organoids neurons and host neurons (Supplementary Fig. 11d). Next, we recorded PSCs in the GFP^−^ host neurons by photostimulating the GFP^+^ fibers from the organoids neurons and found that the illumination resulted in inward currents in GFP^−^ host neurons under voltage-clamp recording conditions at a holding potential of −65 mV and the PSCs were blocked by the application of ionotropic glutamate receptor antagonist kynurenic acid (Supplementary Fig. 11e–g), suggesting a glutaminergic synaptic efferent from the organoids neurons to host neurons. To confirm that the cells recorded electrophysiological signals are host cells but not organoids cells, we infused biocytin into the recorded neuron through an electrode after patch-clamp recording. Immunofluorescence staining showed that biocytin-filled cells did not express EYFP and HN, indicating that they are host neurons but not organoids neurons (Supplementary Fig. 11h).

To further demonstrate the synaptic efferent from the organoids neurons to host neurons in distant targets, we transplanted the organoids derived from H9-CAG-ChR2-EYFP cell line into the junction of the infarct core and the peri-infarct zone of stroke mice, and on day 180 after transplantation, we induced local field potentials (LFPs) in the contralateral cortex and the ipsilateral striatum by photostimulating the fibers projected from the grafted organoids (Fig. 4h). Because of no GFP^+^ neurons in the contralateral cortex and the ipsilateral striatum (Fig. 3), the recordings of LFPs in these areas absolutely reflected the activity of host neurons receiving projections from grafted organoids neurons. As shown in Fig. 4i, j, the illumination of GFP^+^ fibers in both the contralateral cortex and the ipsilateral striatum induced LPFs dose-dependently. Therefore, there is synaptic efferent from the organoids neurons to host neurons, including local and distant targets.

### Restoration of sensorimotor function by transplanting human cerebral organoids after stroke

**Fig. 5:**
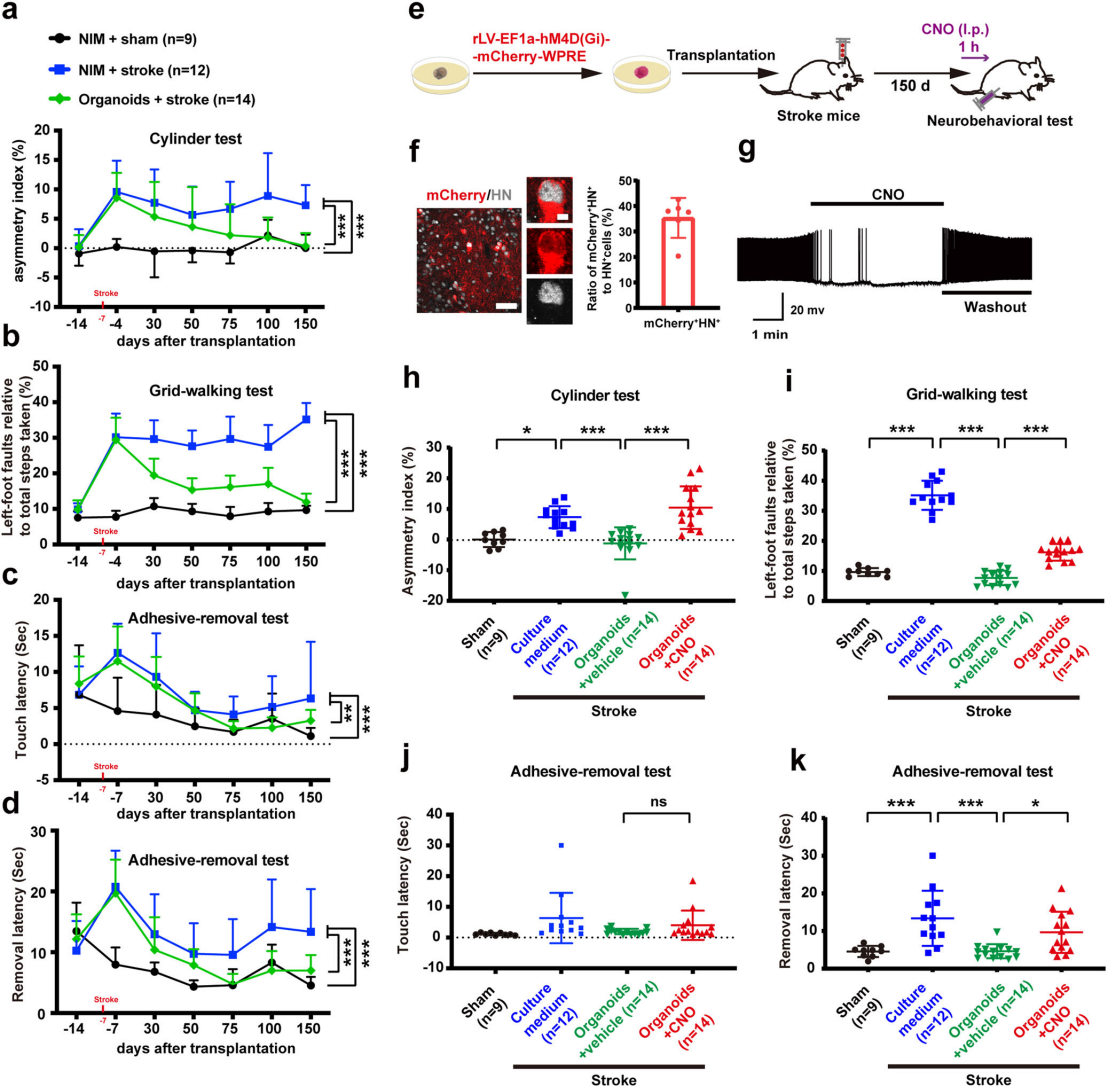
The transplantation of human cerebral organoids promotes functional recovery from stroke in mice. **a** Asymmetry index of forelimbs in the cylinder test (*F*_*2,32*_ = 50.952, ***P < 0.001. ***P < 0.001, NIM/stroke *vs* sham or NIM/stroke *vs* organoids/stroke). **b** Left-foot faults relative to total steps taken in the grid-walking test (*F*_*2,32*_ = 317.196, ***P < 0.001. ***P < 0.001, NIM/stroke *vs* sham or NIM/stroke *vs* organoids/stroke). **c** Latency to touch the sticky dot on left paw in the adhesive-removal test (*F*_*2,32*_ = 27.875, ***P < 0.001. ***P < 0.001, sham *vs* NIM/stroke; **P = 0.002, NIM/stroke *vs* organoids/stroke). **d** Latency to remove the sticky dot on left paw in the adhesive-removal test (*F*_*2,32*_ = 47.683, ***P < 0.001. ^***^P < 0.001, NIM/stroke *vs* sham or NIM/stroke *vs* organoids/stroke). **e** Design of the experiments for **f**–**k**. **f** Image showing mCherry-positive cells in the infarct core (left, scale bar, 50 μm), high magnification images from a selected area in the leftward image (middle, scale bar, 5 μm) and bar graph showing ratio of mCherry^+^/HN^+^ cells to HN^+^ cells (right). *n* = 6 animals. **g** In the neurons expressing hM4Di-mCherry, bath application of CNO completely abolished cell discharge of action potentials and this effect was reversed after CNO washout. **h** Asymmetry index of forelimbs in the cylinder test (*F*_*3,45*_ = 15.679, ***P < 0.001. *P = 0.014, ***P < 0.001). **i** Left-foot faults relative to total steps taken in the grid-walking test (*F*_*3,45*_ = 189.858, ***P < 0.001. ***P < 0.001). **j** Touch latency (*F*_*3,45*_ = 0.062, *P* = 0.062. *P* > 0.05, between groups) in the adhesive-removal test. **k** Remove latency (*F*_*3,45*_ = 9.790, ***P < 0.001. *P = 0.033, ***P < 0.001) in the adhesive-removal test. NIM neural induction medium, HN Human Nuclei. In **a**–**d**, **f** and **h**–**k**, data were presented as mean ± standard deviation.

To assess whether the grafted human cerebral organoids can restore sensorimotor function of stroke mice, we transplanted the organoids into the junction of the infarct core and the peri-infarct zone of mice on day 7 after stroke and assessed sensorimotor functions using cylinder test, grid-walking test, and adhesive-removal test on day −14, −4, 30, 50, 75, 100 and 150 after transplantation. Approximately 93% of grafted animals survived beyond 150 d, which presented no difference from NIM-treated sham or stroke groups (Supplementary Fig. 12a). The body weight of mice showed similar levels in the three groups (Supplementary Fig. 12b). We examined the GFP expression in stroke mice receiving cerebral organoids on day 180 after transplantation and observed survival of transplants (100%, *n* = 14). The cylinder test was used to assess spontaneous forelimb tasks. As shown in Fig. 5a, the NIM-treated stroke mice had a significantly higher asymmetry index than NIM-treated sham mice did (***P < 0.001), and the stroke mice receiving cerebral organoids had a significantly reduced asymmetry index, compared to the stroke mice receiving NIM (***P < 0.001). In the grid-walking test (Fig. 5b), a measure of spontaneous motor defects, foot faults of controlateral forelimb in the NIM-treated stroke mice were significantly more than that in the NIM-treated sham mice (***P < 0.001), and the stroke mice receiving cerebral organoids had significantly reduced foot faults, compared to the stroke mice receiving NIM (***P < 0.001). In the adhesive-removal test (Fig. 5c, d), which is used to assess motor sensory deficits, the latency to touch or remove the adhesive stickers attached to the ipsilateral forepaw in the NIM-treated stroke mice was significantly longer than that in the NIM-treated sham mice (***P < 0.001), and the stroke mice receiving cerebral organoids had significantly reduced touch (**P = 0.002) and removal latency (***P < 0.001), compared to the stroke mice receiving NIM. Moreover, there were similar asymmetry index, foot faults and removal latency between the stroke mice receiving organoids and sham mice in the later stages of organoids transplantation (Fig. 5a, b, d). These data suggest that the transplantation of human cerebral organoids restores sensorimotor function after stroke.

To determine whether the activity of grafted cerebral organoids in stroke mice is necessary for sensorimotor restoration after stroke, we prepared the cerebral organoids from IMR-90–4 iPSCs transduced with rLV-EF1a-hM4D(Gi)-mCherry-WRPE, a recombinant virus vector that silences the activity of infected neurons in the presence of designer drugs (DREADDs) agonist clozapine-N-oxide (CNO), and transplanted them into the junction of the infarct core and the peri-infarct zone of stroke mice. Because systemic CNO injection did not affect sensorimotor behaviors in both sham and NIM-treated stroke mice, compared to vehicle (Supplementary Fig.13), CNO was only applied to grafted organoids group (Fig. 5e–k) on 150 d after transplantation. The hM4D-mCherry^+^ organoids neurons in the infarcted core were shown in Fig. 5f, and the APs of mCherry-expressing organoids neurons were completely abolished by the bath application of CNO and recovered after CNO washout (Fig. 5g), suggesting that hM4Di/CNO system works well. Sensorimotor behaviors were assessed at 1 h after CNO injection (2 mg/kg, i.p.) (Fig. 5e). In the cylinder test, the stroke mice receiving organoids had significantly reduced asymmetry index, compared to the stroke mice receiving NIM (***P < 0.001), and the effect of grafted organoids was reversed by CNO (Fig. 5h). In the grid-walking test (Fig. 5i), the stroke mice receiving organoids displayed significantly reduced foot faults of contralateral forelimb compared to the stroke mice receiving NIM did (***P < 0.001), and CNO treatment significantly weaken the effect of grafted organoids (***P < 0.001). Similarly, CNO treatment also significantly weaken the effect of grafted organoids on removal latency in the adhesive-removal test (*P = 0.033), although it did not affect touch latency (Fig. 5j, k). Thus, the neuronal activity of grafted cerebral organoids is crucial for sensorimotor function restoration after stroke.

## Discussion

Transplantation of neural stem cells to replace lost neuronal function holds promise for stroke recovery, whereas cell therapy is limited due to the inability of dispersed single neurons of implants to truly repair the lost tissue in the infarct core. In this study, we transplanted human cerebral organoids into the junction of the infarct core and the peri-infarct zone of NOD-SCID mice subjected to stroke and found that the grafted cerebral organoids survived well in the infarcted core, differentiated into target neurons, repaired the infarcted core, sent axons to distant brain targets, and integrated into the host neural circuit and thereby restored sensorimotor function of stroke mice.

Unlike organoid transplantation, we found that few cells survived in the infarcted core after the transplantation of dissociated single cells from organoids (Supplementary Fig. 8), suggesting that organoids but not dissociated organoid cells are more likely to survive within the infarcted core. Indeed, several recent studies have transplanted hiPSCs- and mouse iPSCs-derived cortical neural progenitors into the area close to infarct core to investigate whether the treatment improves stroke-induced motor deficits in adult rats and showed that the majority of implants are located in the peri-infarct zone, with only rare cells existed in the infarcted core^31,36^. The difference in fate between organoids and single cells transplantation may be due to that organoids have high self-organizing structures, various cell types, microenvironment and self-renewal capacity^27^, so that they can get more mutual cell-to-cell supports when resisting the adverse environment of the infarcted area.

Moreover, cerebral organoids have distinct differentiation fates in the infarcted core and peri-infarct regions. In the peri-infarct zone, cerebral organoids are mainly composed of mature and immature glutaminergic neurons, with only a small number of astrocytes and oligodendrocytes (Supplementary Fig. 7). Surprisingly, however, more than 65% of organoid cells differentiated into glial cells in the infarcted core (Fig. 2). The different differentiation fate of organoids in peri-infarct zone and in infarcted core may be caused by different inflammatory microenvironments. Extracellular matrix (ECM) is a 3D structure composed of insoluble components^37^ and affects the fate of organoids by providing brain-specific cues and a microfluidic device with periodic flow^38^. Several important cell types are known to contribute to ECM formation and maintenance, such as microglia and vascular endothelial cells^37^. Microglia can be mobilized within an hour and continue to accumulate for over a month after central nervous system injuries^39^. The different fate of organoids in different local environment has left us with an open question that needs to be addressed in the future, that is, whether improving the inflammatory microenvironment of the infarcted core facilitates the survival and differentiation of grafted organoids into functional neurons.

Organoid transplantation favors not only self but also host cells survival in the infarcted core. The cell ratio measurement showed that ~30% of the cells in the infarcted core were HN^−^ on d 180 after organoids transplantation (Fig. 2), suggesting that a considerable number of host cells exist in the infarcted core. However, in contrast to the stroke mice receiving organoid transplantation, in the NIM-treated stroke mice, the infarcted core had become a cavity, and no cells were observed on d 180 after treatment (Supplementary Fig. 9). Stem cell grafts alleviate chronic inflammation^40^, release growth factors and regulate microenvironment through paracrine mechanism in the stroke brain^41^, which may explain the survival of host cells in the infarcted core after organoids transplantation.

Correct and long-distance axon projections from cortex to distant brain targets is crucial for the recovery of sensorimotor functions from stroke. However, whether implants send correct axonal projections to distant brain targets in the infarcted brain remains unknown. Although there are reports that the grafted neurons derived from hiPSCs after transplantation into stroke-injured rat cerebral cortex receive functional synaptic inputs from host neurons^32^, and stroke mice received the combined PSCs-derived neural progenitor cells transduced by luminopsin 3 and luciferase substrate coelenterazine showed enhanced neural network connections in the peri-infarct region^36^, long-distance axon projections from grafted neurons in the infarcted cortex, especially corticospinal projections, have not been identified. So far, several studies reported transplantation of brain organoids intracerebrally and demonstrated survival, differentiation, and vascularization of implants in stroke^42^ or TBI^43^ mouse model, but they did not observe long-distance projections from organoid neurons to distant brain targets, perhaps because they ended their experiments prematurely (4–8 weeks after transplantation). As we all know, it takes a long time for human neurons to mature in the body. We found that stroke mice receiving organoids had a significantly reduced asymmetry on d 75 in the cylinder test (*P = 0.038) and significantly reduced removal latency on d 100 in the adhesive-removal test (*P = 0.039), although they had significantly reduced foot faults on d 30 in the grid-walk test (***P < 0.001), compared to the stroke mice receiving NIM. A recent study^44^ reported that organoids showed LFP responses matching surrounding cortex by visual light stimulation, 3 weeks after organoids transplantation into retroplenial cortex of adult mice, suggesting that xenograft neurons established synaptic connections with the surrounding cortex tissue and received functional input from mouse brain. Thus, in our study, grafted neurons may play a major role in replacement, although we cannot rule out that bystander effect may also contribute to behavioral recovery in the early stage of organoids transplantation into stroke mice. As for which neural circuits contribute to behavioral improvement, it is an open question. Here, we found that grafted organoids neurons in the infarcted cortex can extend long-distance axons to distant brain targets, including callosal, corticostriatal, corticothalamic and corticospinal projections (Fig. 3), and establish synaptic afferent and efferent with local and distant host neurons (Fig. 4, Supplementary Fig. 11), which may be the main reason for functional improvement. Of course, this needs to be confirmed by more experiments in the future.

Moreover, whether the functional recovery from stroke depends on the activity of grafted organoids is an indicator for evaluating the significance of organoid transplantation in the treatment of stroke. To date, however, there is no study to assess the causal relationship between the activity of grafted organoids and functional recovery after stroke, to our knowledge. Using chemogenetics techniques, we demonstrated that the activity of grafted human cerebral organoids is necessary for functional recovery from stroke (Fig. 5).

In sum, grafted cerebral organoids after stroke reconstruct infarcted cortex, send long-distance axon projections to distant brain targets and integrate into the host neural circuits, thereby restoring impaired sensorimotor function, offering a new strategy for reconstructing infarcted tissue and promoting stroke recovery.

## Methods

### Animals

Adult (7- to 8-week-old) male NOD-SCID mice (NOD-Prkdcem26Cd52/Gpt, NCBI number: 19090) were purchased from GemPharmatech (Nanjing, China). Animals were maintained at 20 ± 2 °C and under a 12 h light/dark cycle with food and water provided ad libitum in the animal core facility of Nanjing Medical University. Every effort was made to minimize the number of animals used and their suffering. All animal experiments were approved by the Institutional Animal Care and Use Committee of Nanjing Medical University (approval number: IACUC-1812011) and complied with the Animal Research: Reporting of In vivo Experiments (ARRIVE).

### Human pluripotent stem cells culture

H9-CAG-ChR2-EYFP cell line (a gift from Dr. Su-Chun Zhang, University of Wisconsin-Madison, Madison, USA), hESCs (line H9, WiCell Agreement No. 16-W0060) and hiPSCs (IMR90–4, WiCell Agreement No.17-W0063) were cultured on xeno-free conditions in the Essential 8 medium and passaged every 5–7 days.

### Generation of human cerebral organoids

Generation of human cerebral organoids was performed based on the previously reported protocols^29,30^. Briefly, hiPSCs or hESCs were detached by using dispase to form embryonic bodies (EBs) on day 0 and cultured in the neural induction medium (NIM; DMEM/F12, 1%NEAA, 1% N2). Half of NIM was changed every day from day 2 to day 6. Then, EBs were attached on 6-well plates for the rosette-like neuroepithelial cells (NE) formation from day 7 to day 15. On day 16, the rosettes were gently blown off by a 1 ml pipette tip and resuspended in NIM containing B27. On day 20, cerebral organoids with multiple neural tube structures were obtained. The medium was refreshed every 5–6 days.

### Lentivirus infection of cerebral organoids

Cerebral organoids were collected and placed in a 1.5 ml centrifuge tube, each tube containing about 10 cerebral organoids. In our experiment, there were two kinds of lentivirus (LV) infection solution: solution 1 containing 15 μl LV-EF1a-EGFP, 100 μl NIM, 0.2 μl polybrene and 2.3 μl B27; solution 2 containing 30 μl rLV-EF1a-hM4D(Gi)-mCherry-WPRE, 100 μl NIM, 0.2 μl polybrene and 2.6 μl B27. Both A and B are virus infection enhancers. The prepared lentivirus infection solutions were added into two 1.5 ml centrifuge tubes containing cerebral organoids and incubated at 37 °C for 2 h, and then, transferred to the T12.5 cell culture flask, added to 4 ml NIM. The organoids were transplanted at 10 d after infection with Lentivirus.

### Photothrombotic stroke model and organoids or dispersed organoids cells transplantation

Focal cortical stroke was induced in adult male mice through photothrombosis using a well-established procedure^6,45^. In brief, under 2% isoflurane anesthesia (RWD Life Science), mice were fixed in a stereotaxic apparatus (Model 902, David Kopf Instruments) and their skulls were exposed through a midline incision, cleared of connective tissue, and dried. A cold light source (Z-LITE-Z, World Precision Instruments) attached to a custom-made opaque template giving a 2 mm diameter spot was precisely positioned at two sites (PT-1 and PT-2) independently, following stereotactic coordinates (relative to bregma): (PT-1) anterior-posterior = 0 mm, medial-lateral = + 1.5 mm, dorsal-ventral = 0 mm; (PT-2) anterior-posterior = −1.9 mm, medial-lateral = + 1.5 mm, dorsal-ventral = 0 mm. Rose bengal solution (100 mg/kg, i.p.; Sigma-Aldrich) was intraperitoneally injected, 5 min later, the brain was illuminated with the intensity of 18500 Lux through the exposed intact skull for 15 min. During illumination, rose bengal generated singlet oxygen leading to vascular endothelium damage and occlusion resulting in focal cortical stroke. Following illumination, the skin was surgically glued and the mice were allowed to recover. Control mice received the same surgery and the same dose of rose bengal but did not receive illumination.

One week later, one organoid (~600 μm in diameter and ~70,000 cells in total) or three organoids (~600 μm in diameter and ~200,000 cells in total), single cells (~200,000 cells) dissociated from 3 organoids of similar size with TrypLE, or 3 μl NIM were microinjected into the junction of the infarct core and the peri-infarct zone, using a borosilicate glass capillary. For the microinjections of three organoids, single cells or NIM, the microinjection sites were left, right and inferior to the infarcted core, respectively, and following stereotactic coordinates (relative to bregma): (1) anterior-posterior = 0 mm, medial-lateral = + 0.7 mm, dorsal-ventral = −1.5 mm; (2) anterior-posterior = 0 mm, medial-lateral = + 1.5 mm, dorsal-ventral = −1.7 mm; (3) anterior-posterior = 0 mm, medial-lateral = + 2.5 mm; dorsal-ventral = −2.0 mm. For the microinjections of one organoid, the microinjection site was inferior to the infarcted core following stereotactic coordinates (relative to bregma): anterior-posterior = 0 mm, medial-lateral = + 1.5 mm; dorsal-ventral = −1.7 mm. Core body temperature was measured by a rectal probe and maintained at 37 °C throughout the surgery.

### Assessment of infarct volume

Mice were sacrificed under an overdose of chloral hydrate anesthesia (400 mg/kg, i.p.) and brains were removed. 1 mm coronal slices were made with a vibrating blade microtome (VT1200s, Leica) in ice-cold PBS. Then, Brain slices were incubated with 2% 2,3,5-triphenyl tetrazolium chloride (TTC) at 37 °C for 20 min. Brain sections were captured with a microscope and infarct volume was calculated with Image-J software.

### Recombinant virus and stereotaxic microinjection

Recombinant adeno-associated virus (AAV) AAV-syn-hChR2(E123A)-mCherry (5 × 10^12^ virus particles/ml) was purchased from GeneChem Co., Ltd (Shanghai, China). Recombinant lentivirus rLV-EF1a-hM4D(Gi)-mCherry-WRPE (5 × 10^12^ virus particles/ml) were purchased from BrainVTA Co., Ltd (WuHan, China). Lentivirus LV-EF1a-EGFP (5 × 10^9^ virus particles/ml) were purchased from Nantong Gadgetzan Co., Ltd (NanTong, China) and stored at −80 °C freezer until the day of infusion.

Mice were anesthetized with isoflurane gas (2% induction; 1% maintenance) mixed in oxygen and fixed on a stereotaxic apparatus. A small craniotomy was made targeting the motor cortex with stereotaxic coordinates (anterior-posterior = 0 mm, medial-lateral = + 2.0 mm, dorsal-ventral = −2.0 mm, relative to bregma). Glass capillaries with a tip size of 30 μm in outer diameter were loaded with AAV-syn-hChR2(E123A)-mCherry. Then, 0.35 μl of the virus-containing solution was injected into the motor cortex at an injection rate of 5 nl/s. After injection, injection needles were left in place for an additional 10 min to assure even distribution of the virus and were then slowly withdrawn.

### Immunostaining for organoids and brain slices

Organoids were aspirated with a 1 ml pipette and fixed with 4% PFA for 2 h. Mice were sacrificed under an overdose of chloral hydrate anesthesia (400 mg/kg, i.p.; HuShi) and then were perfused with PBS followed by 4% PFA, and the brains were removed. Organoids or brains were dehydrated with 20% and 30% sucrose solution until completely sunk. Serial sections were obtained by frozen section (CM1950, Leica) at a thickness of 10 μm for organoids and 30 μm for mice brains. Sections were washed by PBS 3 times and treated with 5% donkey serum and 1% triton for 1 h at room temperature. The sections were incubated in primary antibodies, which were prepared with 5% donkey serum and 0.2% Triton at 4 °C overnight. The next day, sections were washed by PBS 3 times and incubated in secondary antibodies diluted in 5% donkey serum at room temperature for 1 h. Finally, the sections were washed with PBS 3 times and mounted by Fluoromount-G. The primary and secondary antibodies are listed in Supplementary Table 1. Images were captured on Nikon Eclipse 80i fluorescence microscope and Zeiss LSM 700B laser confocal microscope. Image J software was used for cell counting, at least 8 random fields were chosen.

### Behavioral test

In this study, the behavioral tests include cylinder test, grid-walking test, and adhesive removal test. Investigators were blinded to group allocation when assessing animal behaviors.

### Cylinder test

Cylinder tasks were performed as previously described in detail with minor modifications. Briefly, each mouse was placed individually in a clear plexiglass cylinder (height, 15 cm; diameter, 10 cm). The mouse would spontaneously rear to a standing position while supporting its weight with either one or both of its forelimbs. A video camera (C525, Logitech) was positioned 20 cm in front of a cylinder to record the free exploration of the mouse for 5 min. The video footage was analyzed offline in slow motion (1/5^th^ real-time speed) by calculating the time (s) during each rear that the animal spent on either its right forelimb, left forelimb, or both forelimbs. Only rears in which both forelimbs could be clearly seen were included. The percentage of time spent on each limb was calculated and these data were used to derive an asymmetry index as follows: (% ipsilateral use) − (% contralateral use)^46^.

### Grid-walking test

The grid-walking test can evaluate the spontaneous movement defects of rodents by using the instinct of mice to explore new environments. To perform this test, each mouse was placed individually on top of an elevated wire grid (length × width × height: 32 × 20 × 50 cm, each square wire meshes size is 1.2 × 1.2 cm) and allowed to walk freely for 5 min. A video camera (C525, Logitech) was positioned 30 cm beneath the wire grid to capture stepping errors (foot faults). The video footage was analyzed offline in slow motion (1/5th real time speed) by a rater who was blind to the treatment groups. If a step did not provide support and the foot went through the grid it was considered a fault. A step was also considered a foot fault if the mouse was resting with the grid at the level of the wrist. The total number of steps for each limb was counted and the ratio between foot fault steps (F) and the total steps (T) of forelimbs was calculated: the number of forelimb foot faults/the total steps of forelimbs × 100%^47^.

### Adhesive removal test

The adhesive removal test was used to evaluate the sensorimotor function, time for a mouse to touch and remove adhesive stickers from the paws was measured as previously described^36,48^. In brief, each mouse was placed individually in a plastic cage to explore the environment for 5 min. Then a sticker (length × width: 0.3 × 0.2 cm) was adhered to on the contralateral forepaw of the stroke mouse to record the whole process of contacting and removing the sticker. Mice were trained three times before surgery. The latency to touch and remove the sticker was counted by video slow-speed playback.

### Electrophysiology

Brains were sectioned with a vibrating blade microtome (VT1200s, Leica) in ice-cold cutting solution containing 2.5 mM KCl, 0.5 mM CaCl_2_, 7 mM MgCl_2_, 1.3 mM NaH_2_PO_4_, 25 mM NaHCO_3_, 1.3 mM Sodium-ascorbate, 0.6 mM Sodium-Pyruvate, 110 mM Choline chloride and 20 mM D-Glucose. The slices were transferred to a recording chamber and were continuously perfused with normal artificial cerebrospinal fluid (ACSF) containing 125 mM NaCl, 2.5 mM KCl, 2 mM CaCl_2_, 1.3 mM MgCl_2_, 1.3 mM NaH2_P_O_4_, 5 mM NaHCO_3_, 10 mM D-Glucose, 1.3 mM Sodium-ascorbate, 0.6 mM Sodium-Pyruvate with 95% O_2_ and 5% CO_2_ at 34 °C for 1 h before recording (flow rate, 4–6 ml/min). The slices were visualized using an upright microscope (Olympus X51W, Nomasky) with a 5 × 0.1 NA or 60 × 1.0 NA objective and infrared and differential interference contrast optics. The fluorescent neurons were visualized under the Olympus microscope equipped with a 60 × water-immersion lens and illuminated with a mercury lamp. For APs recordings, borosilicate glass pipettes were loaded with an intracellular solution containing 4 mM Na-ATP, 5 mM QX-314, 10 mM HEPES, 0.5 mM EGTA, 132.5 mM Cs-Gluconate, 7.5 mM CsCl and 2 mM MgCl_2_. For sPSCs recordings, we used internal solution containing 70 mM K-gluconate, 70 mM KCl, 2 mM NaCl, 2 mM MgCl_2_, 10 mM HEPES, 1 mM EGTA, 2 mM Mg-ATP and 0.3 mM Na2-GTP. EGFP^+^ neurons were clamped at −65 mV, and a series of current steps, going from 0 to 100 pA in 10 pA steps were injected to evoke APs, and sPSCs were recorded at −65 mV.

For ChR2-mediated APs and sPSCs recordings, optogenetic stimulation during recordings was performed using a 470 nm light-emitting diode (LED) source coupled to a fiber guide and a collimator lens to focus the light spot around the recorded neuron or axons. For ChR2-EYFP-expressing grafted cells, blue light pulse (5 ms duration, 10 mW/mm^2^) was illustrated to EYFP^+^ neuron to evoke APs of this neuron or to the EYFP^+^ axons to evoke postsynaptic currents (PSCs) in EYFP^−^ neurons. After recording, the brain slices were washed with PBS, fixed with 4% PFA for immunostaining. To identify the source of GFP^−^ neuron recorded, we filled internal solution containing biocytin into the recorded neuron through microelectrodes after recording and then fixed the slice. For hChR2-mCherry-expressing host cells, blue light pulse (5 ms duration, 10 mW/mm^2^) was illustrated to the mCherry^+^ neuron to evoke APs of this neuron or to mCherry^+^ axons to evoke PSCs in EGFP^+^ graft neurons. In this experiment, kynurenic acid (3 mM) was used to block AMPARs- and NMDARs-mediated EPSCs.

For rLV-EF1a-hM4D(Gi)-mCherry-expressing graft cells, mCherry^+^ neurons were clamped at −65 mV, and spontaneous APs of the neuron were recorded at 0 pA. Then bath application of CNO (5 μM) inhibited AP firing for 10 min and then washed out.

For local field potential (LFP) recordings, microelectrodes (1–2 MΩ) were filled with ACSF. A collimated 470 nm LED was used to photostimulate ChR2-expressing GFP^+^ fibers in contralateral motor cortex and ipsilateral striatum. These fibers were projected from cerebral organoids neurons in the ipsilateral cortex. The slices were stimulated by a single 20 ms light pulse repeated every 4 s for 25 repetitions.

All recordings were acquired with a Digidata 1440 A at 2 kHz (Axon Instruments). Data were collected with pClamp 10.3 software and analyzed using Clamfit 10.3 (Molecular Devices).

### Statistical analysis

Data were presented as mean ± standard deviation (s.d). All data were statistically analyzed using IBM SPSS Statistics 22 and GraphPad Prism 7.0 Comparisons among multiple groups in the time course of behavioral changes were made with two-way repeated-measures ANOVA followed by post hoc Bonferroni test, and other comparisons among multiple groups were made with one-way ANOVA followed by post hoc Bonferroni test (SPSS Statistics 22 software). Comparisons between two groups were made with a two-tailed student’s *t*-test (GraphPad Prism 7.0). The threshold level of significance was set at *p* < 0.05. The sample size was obtained with power analysis and sample size software using a significance level of α = 0.05 with 90% power to detect statistical differences. For animal studies, the sample size was predetermined by our prior experiments. All experiments and data analyses were conducted blinded.

## Supplementary information

Supplementary Information

## Data Availability

All data associated with this study were in the paper or the Supplementary Materials. All reagents were listed in Supplementary Table 1 and reagent requests should be directed to D.-Y.Z. (dyzhu@njmu.edu.cn). All softwares were commercially or freely available and were listed in Supplementary Table 1. All the replicates number of organoids and mice used for immunostaining, behavioral, and electrophysiological experiments were listed in Supplementary Table 2. Additional information required to reanalyze the data in this paper is available from D.-Y.Z. (dyzhu@njmu.edu.cn) upon request.
