# Supplementary material for: Cerebral organoids transplantation repairs infarcted cortex and restores impaired function after stroke
Source: NPJ Regen Med. 2023 May 30;8:27. doi: 10.1038/s41536-023-00301-7 (PMC10229586; doi:10.1038/s41536-023-00301-7)
Supplement: Supplementary file 1 — Supplementary Information [file 41536_2023_301_MOESM1_ESM.pdf]

Supplementary Information

Cerebral organoids transplantation repairs infarcted cortex and restores impaired function after stroke

Shi-Ying Cao[1,3], Di Yang[1], Zhen-Quan Huang[1], Yu-Hui Lin[1], Hai-Yin Wu[1], Lei Chang[1], Chun-Xia Luo[1], Yun Xu[3], Yan Liu[2*], Dong-Ya Zhu[1,2*]

[1]Department of Pharmacology, School of Pharmacy, Nanjing Medical University, Nanjing, 211166, China

[2]Institution of Stem Cells and Neuroregeneration, Nanjing Medical University, Nanjing, 211166, China

[3]Department of Neurology, Affiliated Drum Tower Hospital of Nanjing University, Medical School, Nanjing 210008, China

Dong-Ya Zhu: dyzhu@njmu.edu.cn

*Corresponding authors: dyzhu@njmu.edu.cn or yanliu@njmu.edu.cn

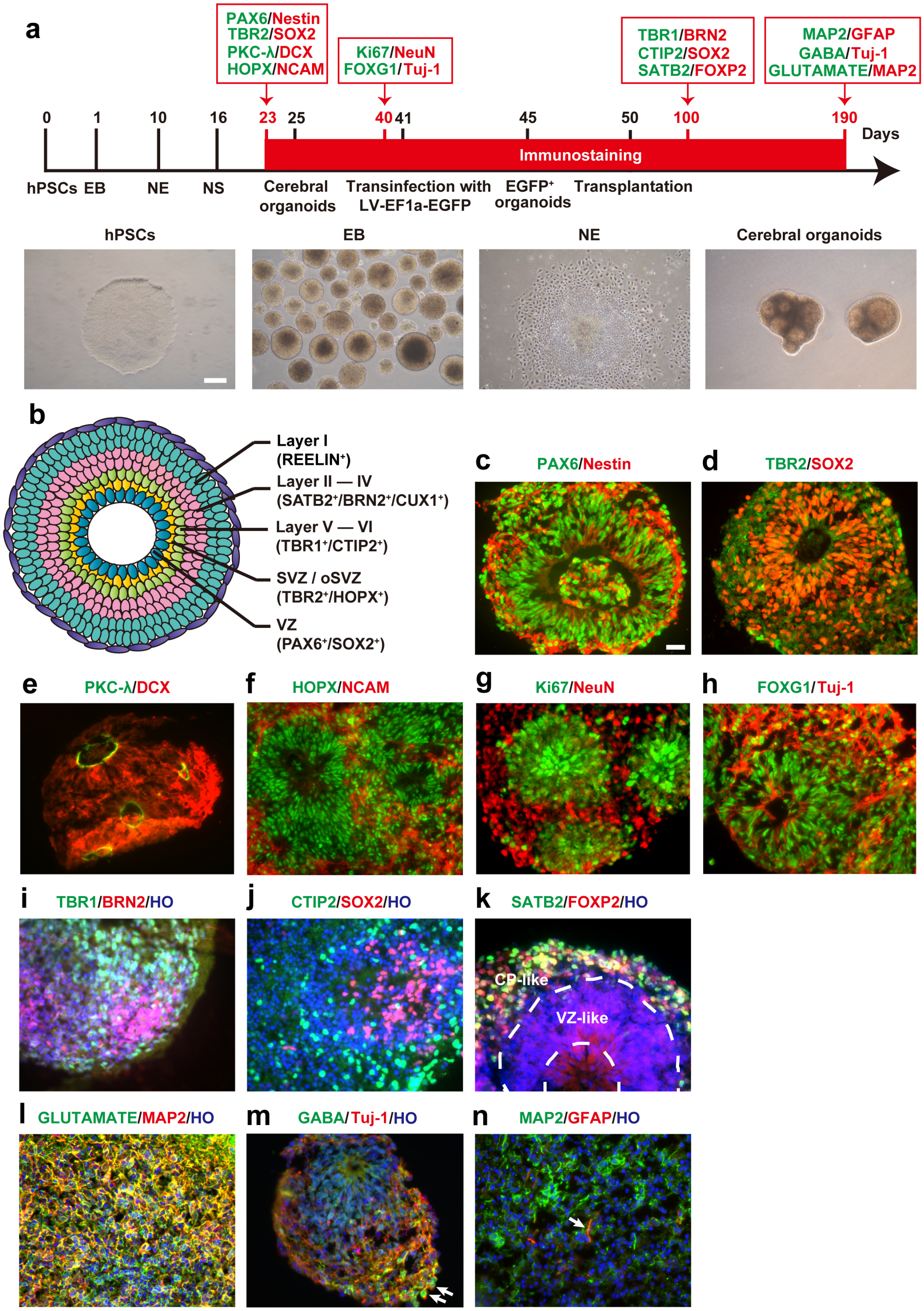
a
PAX6/Nestin
TBR2/SOX2
PKC-λ/DCX
HOPX/NCAM
Ki67/NeuN
FOXG1/Tuj-1
TBR1/BRN2
CTIP2/SOX2
SATB2/FOXP2
MAP2/GFAP
GABA/Tuj-1
GLUTAMATE/MAP2
0 1 10 16 23 25 40 41 45 50 100 190
Days
Immunostaining
hPSCs EB NE NS Cerebral organoids Transinfection with LV-EF1a-EGFP EGFP+ organoids Transplantation
hPSCs
EB
NE
Cerebral organoids
b
Layer I (REELIN+)
Layer II — IV (SATB2+/BRN2+/CUX1+)
Layer V — VI (TBR1+/CTIP2+)
SVZ / oSVZ (TBR2+/HOPX+)
VZ (PAX6+/SOX2+)
c PAX6/Nestin
d TBR2/SOX2
e PKC-λ/DCX
f HOPX/NCAM
g Ki67/NeuN
h FOXG1/Tuj-1
i TBR1/BRN2/HO
j CTIP2/SOX2/HO
k SATB2/FOXP2/HO
CP-like
VZ-like
l GLUTAMATE/MAP2/HO
m GABA/Tuj-1/HO
n MAP2/GFAP/HO

Supplementary Figure 1. Generation of human cerebral organoids *in vitro*.

(**a**) Schematic showing the differentiation, timeline of immunostaining, transduction with LV and transplantation of hPSCs-derived human cerebral organoids, example images of each stage were shown. Scale bar, 250 μm. (**b**) Schematic illustrating the laminar organization and cellular composition of human cerebral organoids. (**c-f**) Representative images showing cerebral organoids stained with the dorsal forebrain progenitor marker PAX6 and neural stem cells marker Nestin (**c**), VZ marker SOX2 and intermediate progenitor marker TBR2 (**d**), the apical marker protein kinase C-λ (PKC-λ) and immature neurons marker doublecortin (DCX) (**e**), and oRGs marker HOPX and neural cell adhesion molecule marker NCAM (**f**) on d 23 after differentiation. (**g** and **h**) Representative images showing cerebral organoids stained with mature neuron marker NeuN and proliferation marker Ki67 (**g**), forebrain marker FOXG1 and neuron marker Tuj-1 (**h**) on d 40 after differentiation. (**i-k**) Representative images showing cerebral organoids stained with deep-layer markers TBR1, FOXP2, and CTIP2, and up-layer markers BRN2 and SATB2 on d 100 after differentiation. (**l-n**) On d 190 after differentiation, cerebral organoids were mainly composed of glutamatergic neurons (Glutamate/MAP2), with only a few GABAergic neurons (GABA) and astrocytes (GFAP). Scale bar, **c**-**n**, 25 μm.

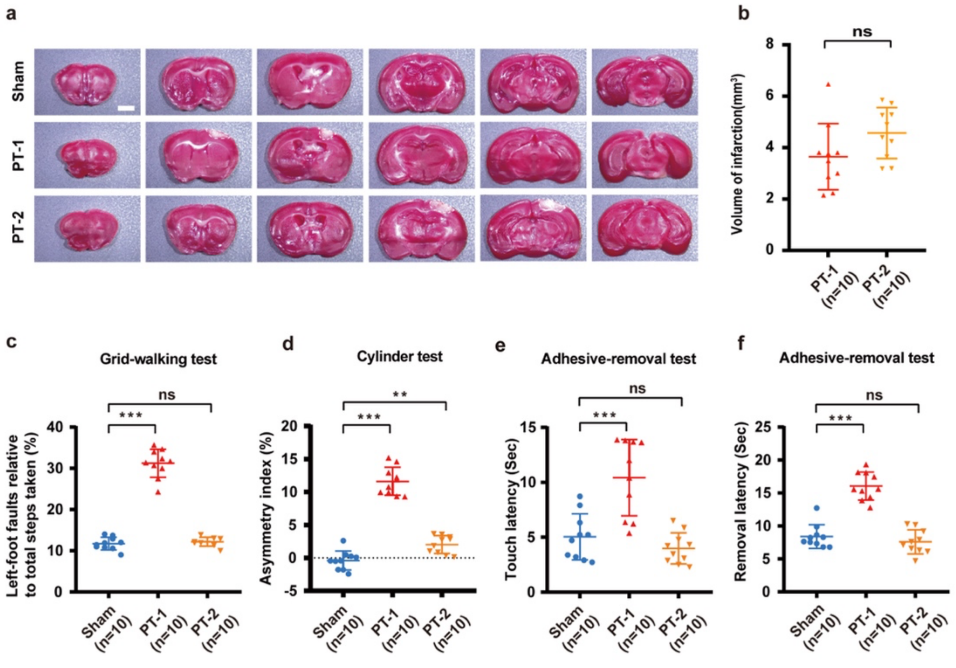

Supplementary Figure 2. Behavioral modifications after photothrombotic stroke in the forelimb motor cortex or parietal cortex.

(a) Representative of TTC–stained slices. Scale bar, 2 μm. **(b)** Infarct volume of mice subjected to photothrombotic stroke in the forelimb motor cortex or parietal cortex (*t*-test, P = 0.089). **(c)** Left-foot faults relative to total steps taken in the grid-walking test (One-way ANOVA, $F_{2,27} = 246.3$, $^{***}P < 0.001$). **(d)** Asymmetry index of forelimbs in the cylinder test (One-way ANOVA, $F_{2,27} = 144.3$, $^{**}P = 0.009$, $^{***}P < 0.001$). **(e)** Touch latency (One-way ANOVA, $F_{2,27} = 19.35$, $^{***}P < 0.001$) in the adhesive-removal test. **(f)** Remove latency (One-way ANOVA, $F_{2,27} = 59.49$, $^{***}P < 0.001$) in the adhesive-removal test. n = 10 animals for each group. In **b-f**, data were presented as mean ± standard deviation.

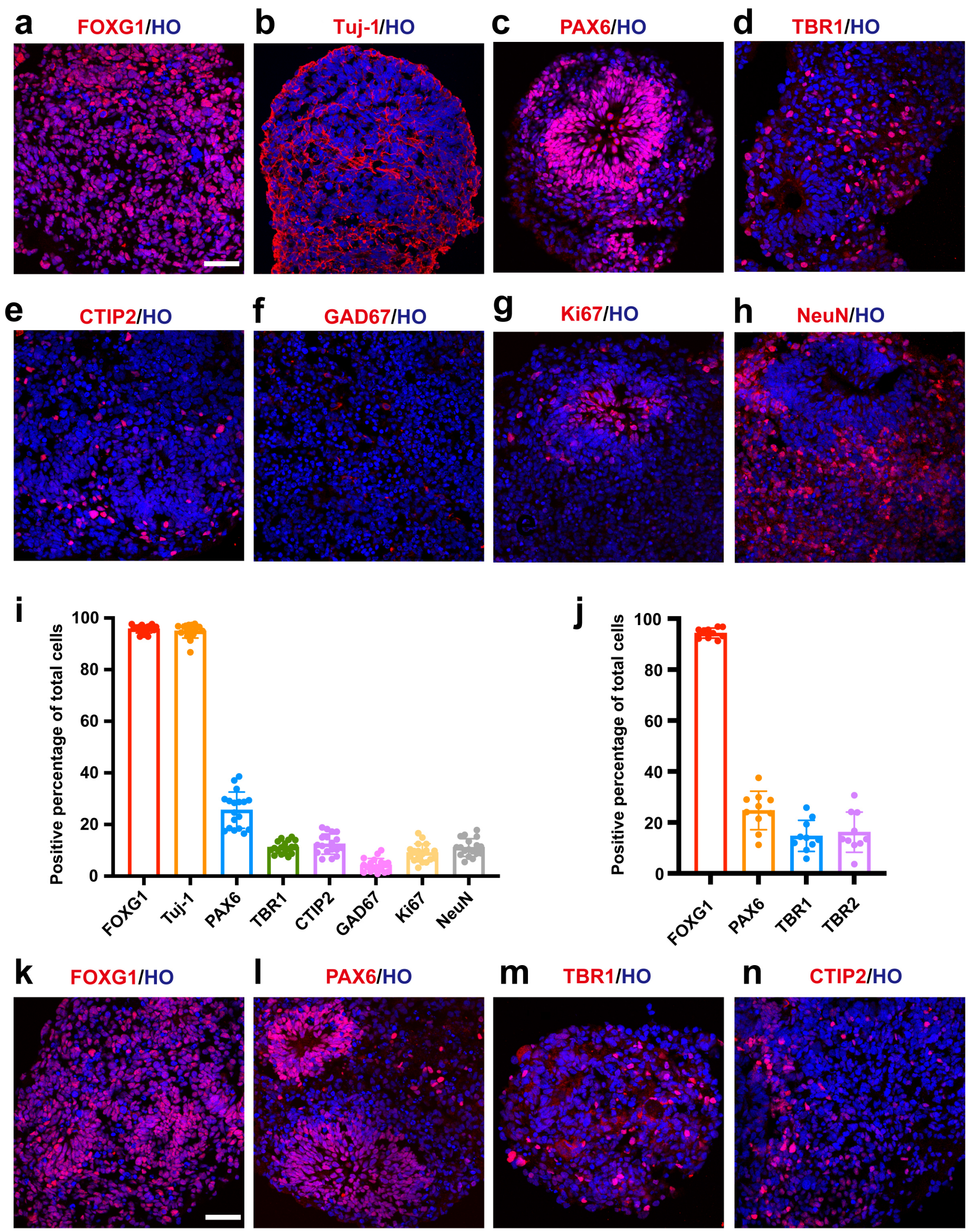

Supplementary Figure 3. The cell composition of cortical organoids differentiated for 50 days in vitro.

(a-h) Representative images showing cerebral organoids derived from hESCs stained with the forebrain marker FOXG1**(a)**, neuron marker Tuj-1(**b**), dorsal forebrain

progenitor marker PAX6 **(c)**, deep-layer (V-VI) cortical marker TBR1**(d)** and CTIP2 **(e)**, GABAergic neuron marker GAD67 (**f**), cell proliferation marker Ki67 (**g**) and mature neuron marker NeuN (**h**) on 50 d after differentiation *in vitro*. Scale bar, **a**-**h**, 50 μm. (**i**) Bar graph showing the proportion of FOXG1-, Tuj-1-, PAX6-, TBR1-, CTIP2-, GAD67-, Ki67-, and NeuN-positive cells on 50 d after differentiation. *n*=18 organoids. More than 10,000 cells from random fields were manually counted in each condition. **(j)** Bar graph showing the proportion of FOXG1-, PAX6-, TBR1-, CTIP2-, cells derived from IMR-90-4 on 50 d after differentiation. *n*=10 organoids. More than 5,000 cells from random fields were manually counted in each condition. **(k-n)** Representative images showing cerebral organoids derived from IMR-90-4 stained with the forebrain marker FOXG1**(k)**, dorsal forebrain progenitor marker PAX6 **(l)**, deep-layer (V-VI) cortical marker TBR1 **(m)** and CTIP2 **(n)** on 50 d after differentiation. *n*=10 organoids. More than 5,000 cells from random fields were manually counted in each condition. Scale bar, **k**-**n**, 50 μm. In **i** and **j**, data were presented as mean ± standard deviation.

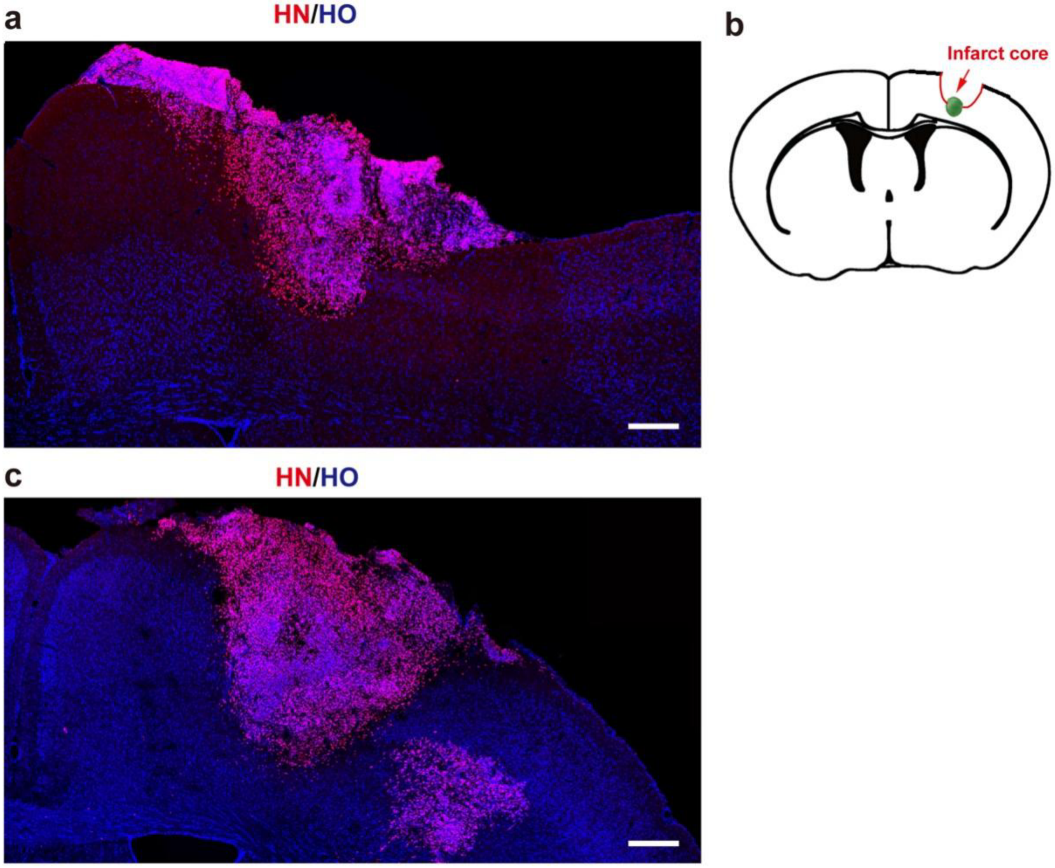

Supplementary Figure 4. Immunostaining showing the repair of infarcted cavity on 60 d after transplantation of one or three organoids.

(**a**) Representative images showing the repair of infarcted cavity on d 60 after transplantation of one organoid. Scale bar, 10 μm. **(b)** Diagram showing the site of one organoid transplant. (**c**) Representative images showing the repair of infarcted cavity on d 60 after transplantation of three organoids. Scale bar, 10 μm.

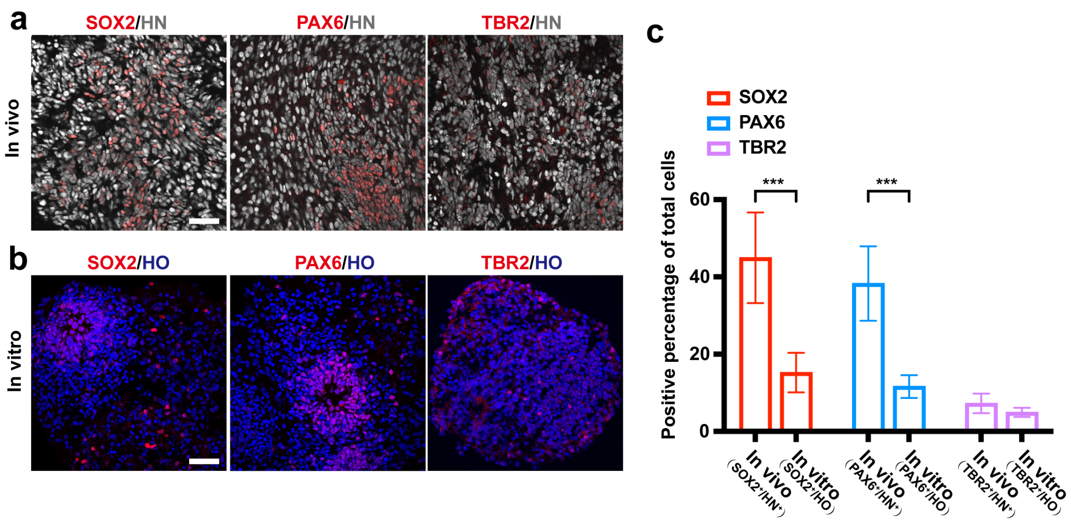

Supplementary Figure 5. Comparison of cell comparison of cortical organoids cultured in vitro for 95 days with cultured in vitro for 50 days and then transplanted into brain for 45 days.

(**a-b**) Representative images showing the neuronal progenitor markers SOX2, dorsal forebrain progenitor marker PAX6, and intermediate progenitor marker TBR2 in the organoids cultured in vitro for 50 days and then transplanted into brain for 45 days (**a)** or in the organoids cultured in vitro for 95 days **(b)**. Scale bar, **a**-**b**, 50 µm. (**c**) Bar graph showing cell populations of two kinds of organoids. n = 6 animals for **a** and n = 18 organoids for **b**. More than 10,000 cells from random fields were manually counted in each condition. In **c**, data were presented as mean ± standard deviation.

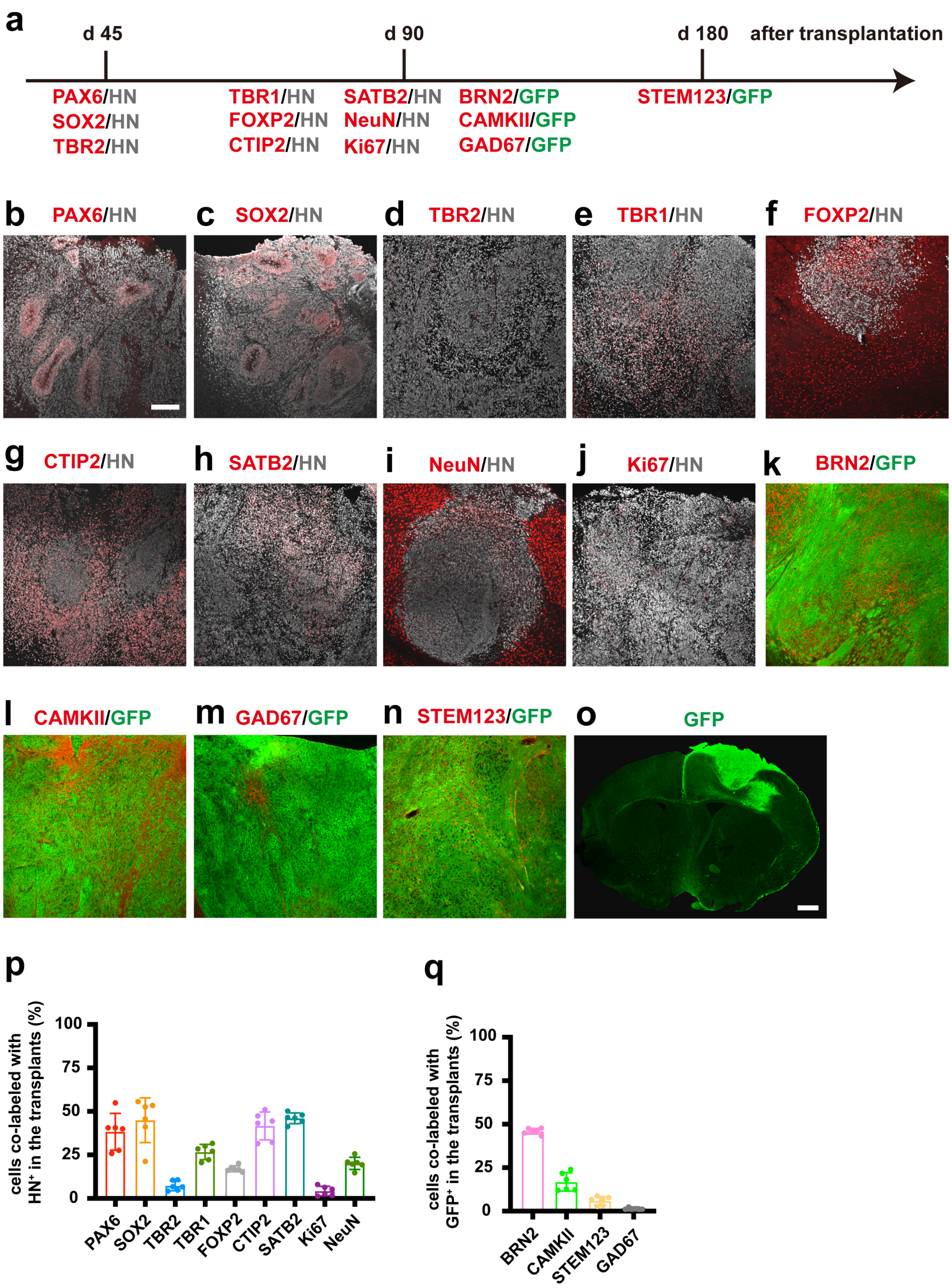

Supplementary Figure 6. The survival and differentiation of cortical organoids after transplantation into the cortex of stroke mice.

(a) Schematic showing the immunostaining timeline after hESCs-derived human cerebral

organoids transplantation. **(b-n)** Representative images showing grafts stained with dorsal forebrain progenitor marker PAX6 **(b)** neuronal progenitor markers SOX2 **(c)**, intermediate progenitor marker TBR2 **(d)**, deep-layer (V-VI) cortical marker TBR1, FOXP2 and CTIP2 and (**e-g**), upper layers (II-IV) cortical marker SATB2 **(h)**, mature neuron marker NeuN (**i**), cell proliferation marker Ki67 (**j**). **(k-n)** Representative images showing GFP^+ transplants stained with upper layers (II-IV) cortical marker BRN2 **(k)**, pyramidal neurons marker CaMKII **(l)**, GABAergic neuron marker GAD67 **(m)** and human astrocytes marker STEM123 **(n)**. Scale bar, **b-n**, 50 µm. **(o)** Immunostaining for GFP^+ graft 150 d in stroke mice transplanted with human cerebral organoids. Scale bar, 150 µm. **(p-q)** Bar graph showing the proportion of PAX6-, SOX2-, TBR2-, TBR1-, FOXP2-, CTIP2-, SATB2-, Ki67-, NeuN- **(p)** and BRN2-, CAMKII-, STEM123-, GAD67- **(q)** positive cells after differentiation. *n* = 6 animals. More than 10,000 cells from random fields were manually counted in each condition. In **p** and **q**, data were presented as mean ± standard deviation.

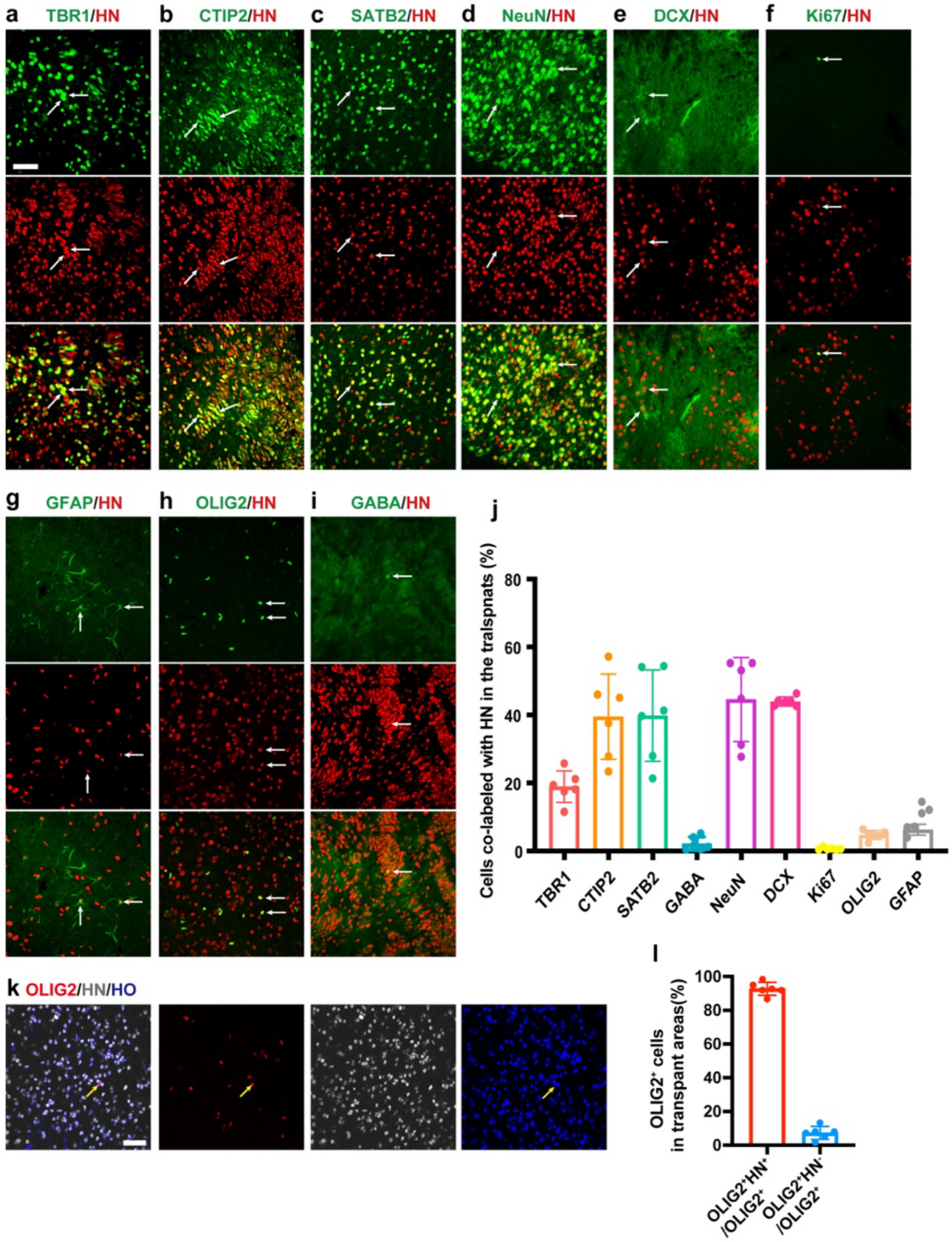

Supplementary Figure 7. Differentiation of human cerebral organoids on d 180 after transplantation into the cortex of stroke mice.

(**a-i**) Representative images showing deep-layer (V-VI) cortical marker TBR1 and CTIP2 (**a** and **b**), upper layers (II-IV) cortical marker SATB2 (**c**), mature neuron marker NeuN

(**d**), immature neuron marker DCX (**e**), cell proliferation marker Ki67 (**f**), astrocyte marker GFAP (**g**), oligodendrocyte marker OLIG2 (**h**) and GABAergic neuron marker GABA (**i**) in the grafted human cerebral organoids (HN^+ cells) 180 d after transplantation. Scale bar, **a**-**i**, 50 μm. (**j**) Bar graph showing the proportion of TBR1-, CTIP2-, SATB2-, NeuN-, DCX-, Ki67-, OLIG2-, GFAP- and GABA-positive cells in the grafts 180 d after transplantation. (**k**) Representative images showing HN-positive and HN-negative OLIG2 cells on d 180 after transplantation. Scale bar, 50 μm. (**l**) Bar graph showing the proportion of OLIG2 derived from host and grafted organoids. $n = 6$ animals. More than 11,000 cells from random fields were manually counted in each condition. In **j** and **l**, data were presented as mean ± standard deviation.

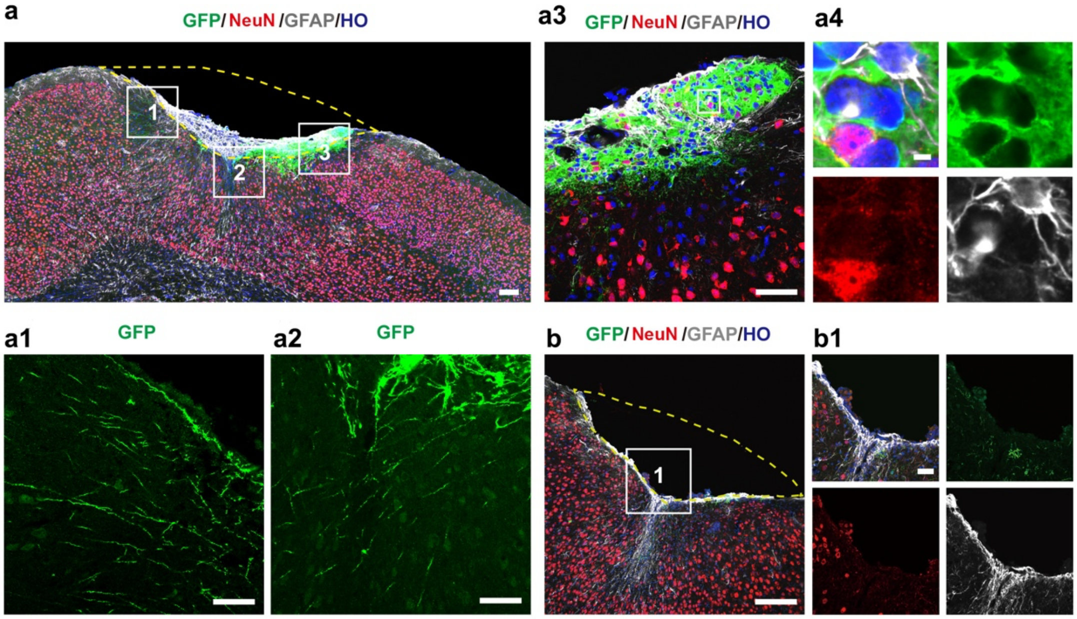

Supplementary Figure 8. Dispersed cerebral organoids cells transplantation cannot repair infarcted tissue on d 150 after transplantation.

Imaging showing the survival of dispersed organoids cells after transplantation into the junction area between the infarct core and peri-infarct zone in **(a)** animal A and **(b)** animal B on d 180 after transplantation (scale bar, 200 μm). a1 and a2: high-magnification images of GFP^+ fibers from the selected areas 1 and 2 in image a respectively (scale bar, 50 μm). a3: A high-magnification image from the selected area 3 in image a (scale bar, 50 μm). a4: High-magnification images from the selected area in image a3 (scale bar, 5 μm). b1: High-magnification images from the selected area in image b (scale bar, 50 μm).

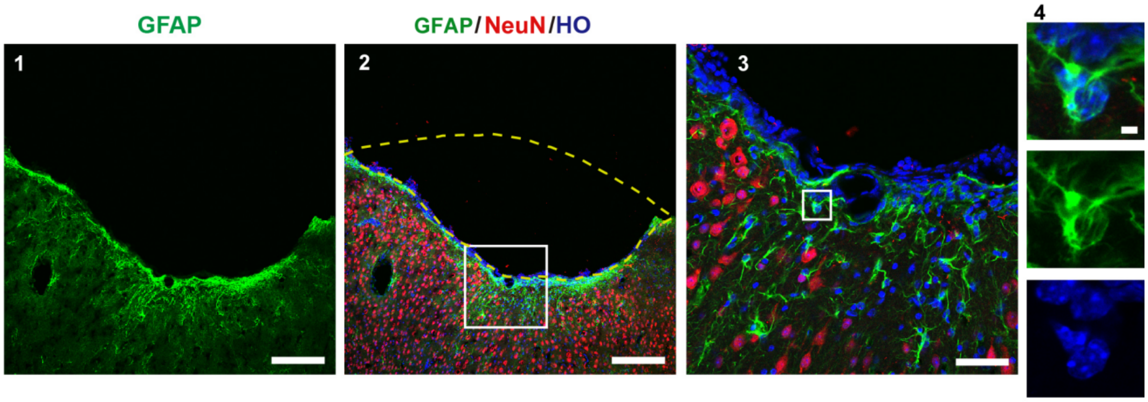

Supplementary Figure 9. Permanent loss of brain tissue within the infarct core on d 180 after stroke.

1: Image showing glial scar at the border of the infarct (scale bar, 200 µm). 2: Image showing infarcted cavity (scale bar, 200 µm). 3: High-magnification image from the selected area in image 2 (scale bar, 50 µm). 4: High-magnification images from the selected area in image 3 (scale bar, 5 µm).

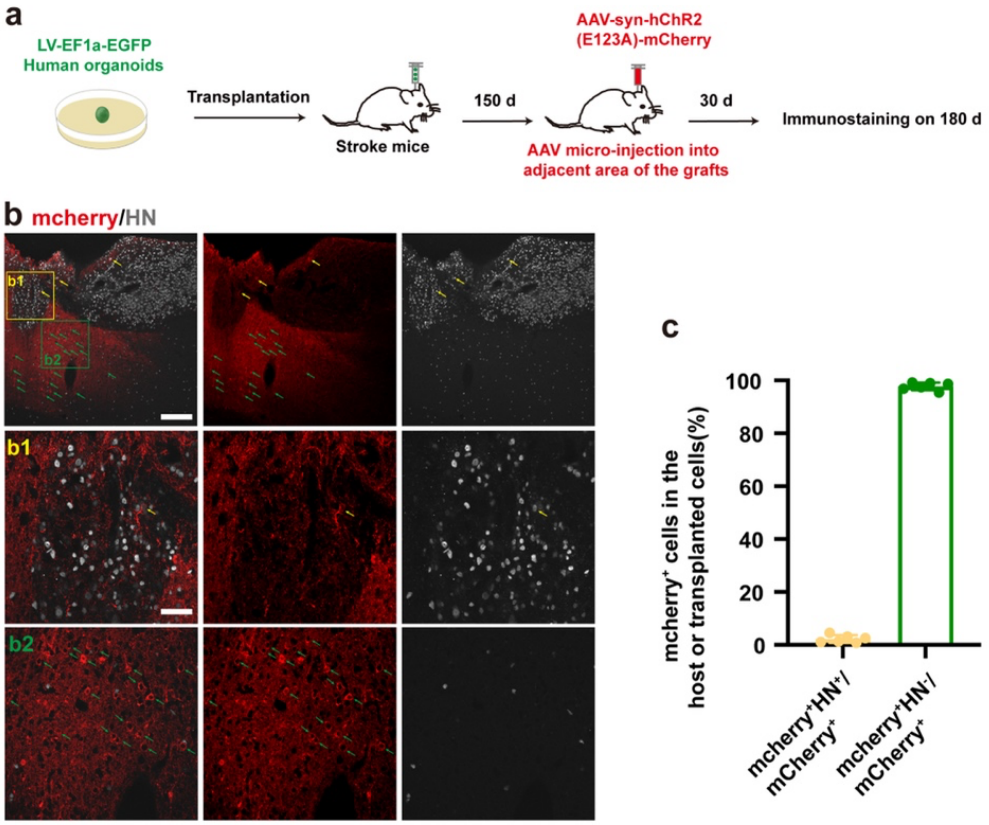

Supplementary Figure 10. Immunostaining for coronal section at 30 d after AAV-syn-hChR2(E123A)-mCherry microinjection.

(a) Schematic showing the timeline of transplantation, AAV microinjection, and immunostaining. **(b)** A representative image showing mCherry^{+} cells co-labeled with HN (yellow arrow) or not co-labeled with HN (green arrow). The yellow and green boxed areas in b were shown in high magnification in b1 and b2 respectively. scale bar, 200 μm (**b**) and 50 μm (**b1**, **b2**). **(c)** Bar graph showing the proportion of mCherry^{+} cells derived from host (HN-negative) and grafted organoids (HN-positive), data were presented as mean ± standard deviation.

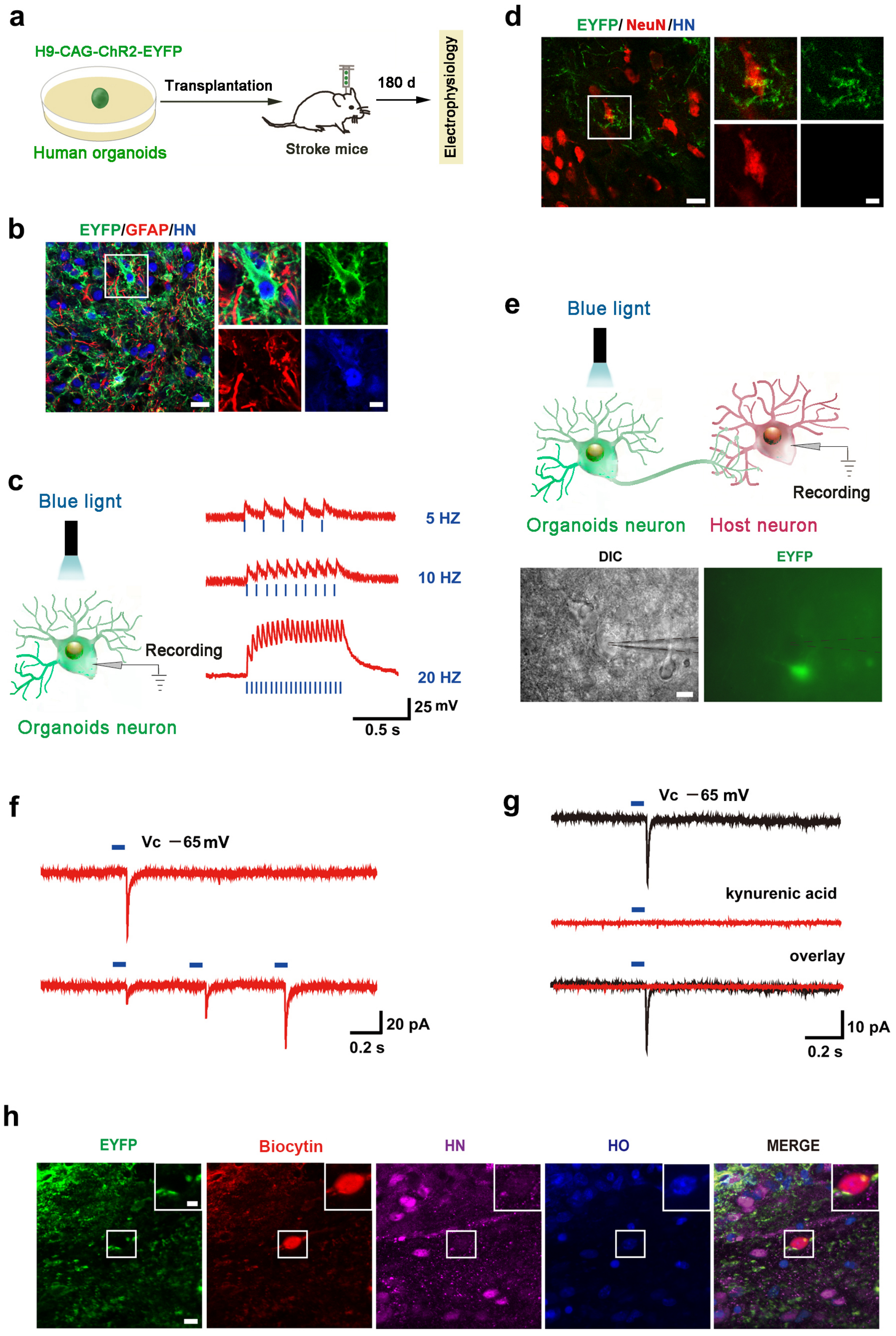
a
H9-CAG-ChR2-EYFP
Transplantation
180 d
Electrophysiology
Human organoids
Stroke mice
d
EYFP/ NeuN /HN
b
EYFP/GFAP/HN
e
Blue lignt
Recording
Organoids neuron
Host neuron
DIC
EYFP
c
Blue lignt
5 HZ
10 HZ
Recording
20 HZ
25 mV
0.5 s
Organoids neuron
f
Vc −65 mV
20 pA
0.2 s
g
Vc −65 mV
kynurenic acid
overlay
10 pA
0.2 s
h
EYFP
Biocytin
HN
HO
MERGE

Supplementary Figure 11. Host neurons receive synaptic input from the grafted organoids neurons.

(**a**) Design of the experiments for **b-h**. Human cerebral organoids were generated from H9-CAG-hChR2-EYFP cell line. (**b**) A representative image showing that $EYFP^+$ cells are co-labeled with HN (left, scale bar, 20 μm) and high magnification images from a selected area in the leftward image (right, scale bar, 5 μm). (**c**) Diagram (left) showing the strategy to evoke APs in the organoids neuron and APs traces (right) evoked by blue photo stimuli pulses (470 nm, 10 mW/mm^2, 5 ms duration) of various frequencies on the ChR2-EYFP-expressing organoids neuron. (**d**) A representative image showing $EYFP^+$ fibers extending to $NeuN^+/HN^-$ host neurons (left, scale bar, 20 μm) and high magnification images from a selected area in the leftward image (right, scale bar, 5 μm). (**e**) Diagram (upper) showing the strategy to test whether host neurons receive synaptic input from organoids neurons and images (lower) showing whole-cell patch-clamp recordings from host neurons (GFP^-). Black dotted lines indicate the placement of the patch-clamp electrode. Scale bar, 20 μm. (**f**) Representative traces of PSCs evoked by 1 or 3 blue photo stimuli pulses at a holding potential of -65 mV. (**g**) Representative traces of PSCs evoked by 1 blue photo stimuli pulse with or without kynurenic acid. In **f** and **g**, n = 19 neurons from 6 mice, in which, 6 neurons were responsive to photostimulation. (**h**) Representative images showing the host neuron stained by biocytin, in which, $biocytin^+$ neuron was EYFP- and HN-negative. Biocytin was filled into the neuron through electrode after patch-clamp recording (left, scale bar, 10 μm) and high magnification

images from a selected area in the leftward image (right, scale bar, 5 μm).

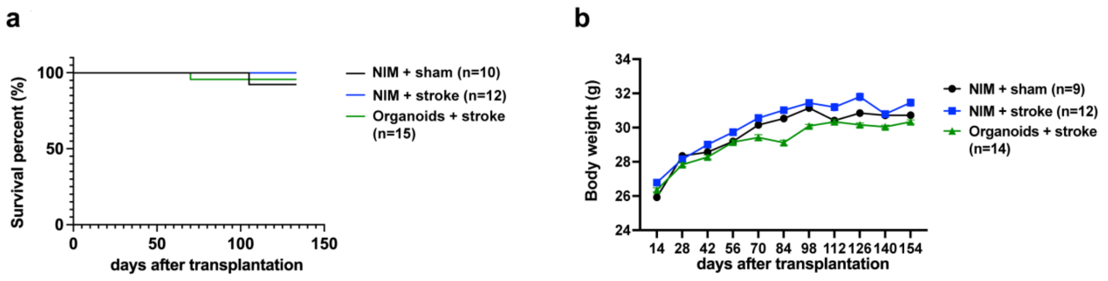

Supplementary Figure 12. Survival percentage and body weight in NOD-SCID mice.

(a) Survival percentage of NOD-SCID mice in three groups. **(b)** Body weight of NOD-SCID mice in three groups, mice were excluded when mortality occurred, data were presented as mean ± standard deviation.

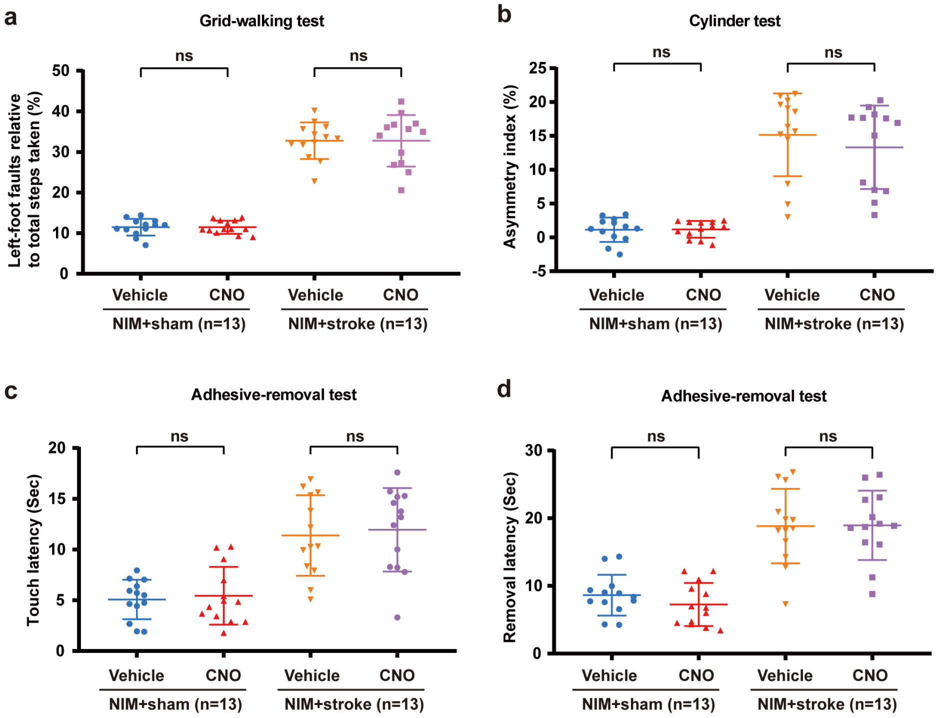

Supplementary Figure 13. Systemic injection of CNO does not affect sensorimotor behaviors.

(a) Left-foot faults relative to total steps taken in the grid-walking test (One-way ANOVA, $F_{3,48}$ = 116.6, $P > 0.05$, between groups). **(b)** Asymmetry index of forelimbs in the cylinder test (One-way ANOVA, $F_{3,48}$ = 37.33, $P > 0.05$, between groups). **(c)** Touch latency (One-way ANOVA, $F_{3,48}$ = 16.08, $P > 0.05$, between groups) in the adhesive-removal test. **(d)** Remove latency (One-way ANOVA, $F_{3,48}$ = 27.62, $P > 0.05$, between groups) in the adhesive-removal test. n = 13 animals for each group. In **a-d**, data were presented as mean ± standard deviation.

Supplementary Table 1. Key resources table

REAGENT or RESOURCE	SOURCE	IDENTIFIER
**Antibodies**		
Mouse monoclonal anti-BRN2	Santa Cruz Biotechnology	Cat# sc-393324; RRID: AB_2737347
Mouse monoclonal anti-CAMKII	Cell Signaling Technology	Cat# 50049 RRID: AB_2721906
Rat monoclonal anti-CTIP2	Abcam	Cat# ab18465; RRID: AB_2064130
Rabbit polyclonal anti-DCX	Cell Signaling Technology	Cat# 4604; RRID: AB_561007
Rabbit polyclonal anti-FOXP2	Abcam	Cat# ab16046; RRID: AB_2107107
Rabbit polyclonal anti-FOXG1	Abcam	Cat# ab18259; RRID: AB_732415
Rabbit polyclonal anti-GABA	Sigma-Aldrich	Cat# A2052; RRID: AB_477652
Mouse monoclonal anti-GAD67	Merck	Cat# MAB5406; RRID: AB_2278725
Rabbit polyclonal anti-GFAP	Agilent	Cat# Z0334; RRID: AB_10013382
Rabbit polyclonal anti-GFP	Millipore	Cat# AB3080P; RRID: AB_2630379
Chicken polyclonal anti-GFP	Millipore	Cat# AB16901; RRID: AB_90890
Rabbit polyclonal anti-GLUTAMATE	Sigma-Aldrich	Cat# G6642; RRID: AB_259946
Mouse monoclonal anti-Human Nuclei	Millipore	Cat# MAB1281; RRID: AB_94090
Rabbit polyclonal anti-HOPX	Sigma-Aldrich	Cat# HPA030180; RRID: AB_10603770
Rabbit polyclonal anti-Ki67	Thermo Fisher Scientific	Cat# PA5-19462; RRID: AB_10981523
Mouse monoclonal anti-MAP2	Sigma-Aldrich	Cat# M1406; RRID: AB_477171
Rabbit polyclonal anti-NeuN	Millipore	Cat# ABN78; RRID: AB_10807945
Mouse monoclonal anti-NeuN	Millipore	Cat# MAB377; RRID: AB_2298772
Goat polyclonal anti-Nestin	Santa Cruz Biotechnology	Cat# sc-21247; RRID: AB_650014
Mouse monoclonal anti-Human NCAM	Santa Cruz Biotechnology	Cat# sc-106; RRID: AB_627128
Rabbit polyclonal anti-PAX6	Covance	Cat# PRB-278P; RRID: AB_291612
Mouse monoclonal anti-PKC-λ	BD Biosciences	Cat# 610207; RRID: AB_397606
Mouse monoclonal anti-Human SATB2	Abcam	Cat# ab51502; RRID: AB_882455

Rabbit monoclonal anti-SATB2	Abcam	Cat# ab92446; RRID: AB_10563678
Goat polyclonal anti-Human SOX2	R and D Systems	Cat# AF2018; RRID: AB_355110
Mouse monoclonal anti-STEM121	Takara Bio	Cat# AB-121-U-050; RRID: AB_2632385
Mouse monoclonal anti-STEM123	Takara Bio	Cat# Y40420; RRID: AB_2833249
Mouse monoclonal anti-Synaptophysin	Thermo Fisher Scientific	Cat# 14-6525-80; RRID: AB_10670424
Rabbit polyclonal anti-TBR1	Abcam	Cat# ab31940; RRID: AB_2200219
Rabbit polyclonal anti-TBR2	Abcam	Cat# ab23345; RRID: AB_778267
Mouse monoclonal anti-Tuj-1	Sigma-Aldrich	Cat# T8660; RRID: AB_477590
Rabbit polyclonal anti-Tuj-1	Covance	Cat# PRB-435P-100; RRID: AB_291637
Donkey anti-Mouse IgG (H+L) Highly Cross-Adsorbed Secondary Antibody, Alexa Fluor 488	Thermo Fisher Scientific	Cat# A-21202; RRID: AB_141607
Donkey anti-Rabbit IgG (H+L) Highly Cross-Adsorbed Secondary Antibody, Alexa Fluor 488	Thermo Fisher Scientific	Cat# A-21206; RRID: AB_2535792
Donkey anti-Goat IgG (H+L) Highly Cross-Adsorbed Secondary Antibody, Alexa Fluor 488	Thermo Fisher Scientific	Cat# A-11055; RRID: AB_2534102
Goat anti-Chicken IgY (H+L) Highly Cross-Adsorbed Secondary Antibody, Alexa Fluor 488	Thermo Fisher Scientific	Cat# A-11039; RRID: AB_2534096
Donkey anti-Mouse IgG (H+L) Highly Cross-Adsorbed Secondary Antibody, Alexa Fluor 546	Thermo Fisher Scientific	Cat# A10036; RRID: AB_2534012
Donkey anti-Rabbit IgG (H+L) Highly Cross-Adsorbed Secondary Antibody, Alexa Fluor 546	Thermo Fisher Scientific	Cat# A10040; RRID: AB_2534016
Donkey anti-Rat IgG (H+L) Highly Cross-Adsorbed Secondary Antibody, Alexa Fluor 594	Thermo Fisher Scientific	Cat# A-21209; RRID: AB_2535795
Donkey anti-Goat IgG (H+L) Highly Cross-Adsorbed Secondary Antibody, Alexa Fluor 546	Thermo Fisher Scientific	Cat# A-11056; RRID: AB_142628

Donkey anti-Mouse IgG (H+L) Highly Cross-Adsorbed Secondary Antibody, Alexa Fluor 647	Thermo Fisher Scientific	Cat# A-31571; RRID: AB_162542
Goat anti-Chicken IgG (H+L) Highly Cross-Adsorbed Secondary Antibody, Alexa Fluor 647	Thermo Fisher Scientific	Cat# A21449; RRID: AB_1500594
Hoechst 33342	Cell Signaling Technology	Cat# 4082; RRID: AB_10626776
**Bacterial and Virus Strains**		
AAV-syn-hChR2(E123A)-mCherry	GeneChem	N/A
rLV-EF1a-hM4D(Gi)-mCherry-WRPE	BrainVTA	Cat# LV-0378
LV-EF1a-EGFP	Nantong Gadgetzan	N/A
**Chemicals, Peptides, and Recombinant Proteins**		
B27 Supplement	Life Technologies	Cat# 17504044
Biocytin	Sigma-Aldrich	Cat# 576-19-2
Choline chloride	Sigma-Aldrich	Cat# C7527-100G
Cesium-gluconate	Hello-bio	Cat# HB4822
CsCl	Sigma-Aldrich	Cat# 7647-17-8
Donkey Serum	Milipore	Cat# S30-KC
Dispase	Gibco	Cat# 17105041
DMEM/F12	Life Technologies	Cat# C11330
DMH-1	Tocris	Cat# 4126
DMSO	Sigma-Aldrich	Cat# D8418
Essential 8 Basal medium	Life Technologies	Cat# A14666SA
Essential 8 Supplement	Life Technologies	Cat# A1517001
EDTA	Lonza	Cat# 17-711E
EGTA	Tocris	Cat# 2807
FBS	Life Technologies	Cat# 0099-141
Glucose	Diamond	Cat# A1001880500
HEPES	BBI Lifescience	Cat# A600264-0250
Kynurenic acid	Tocris	Cat# 223

K-Gluconate	Sigma-Aldrich	Cat# G4500
Mg-ATP	Sigma-Aldrich	Cat# 74804-12-9
Na-ATP	Sigma-Aldrich	Cat# A6419
Na_2-ATP	Sigma-Aldrich	Cat# 34369-07-8
$NaHCO_3$	BBI Life Science	Cat# A610482-0500
Non-essential amino acids solution（NEAA）	Gibco	Cat# 11140
N2	Gibco	Cat# 17105041
PBS tablets	Medicago	Cat# 09-9400-100
QX-314	Tocris	Cat# 2313
Rose Bengal	Sigma-Aldrich	Cat# 330000-5G
Rock inhibitor	Stem cell	Cat# 72304
SB431542	Tocris	Cat# 1614
Sodium-L-ascorbate	Sigma-Aldrich	Cat# A7631-25G
Sodium-Pyruvate	Sigma-Aldrich	Cat# P5280-25G
Sucrose	BIOFROXX	Cat# 1245GR500
TrypLE	Thermo Fisher Scientific	Cat# 12604013
2% 2,3,5-triphenyl tetrazolium chloride (TTC)	Sigma-Aldrich	Cat# T8877
Vitronectin	Life Technologies	Cat# A14700
**Experimental Models: Cell Lines**		
H9 CAG-ChR2-EYFP	A gift from Dr. Su-Chun Zhang	N/A
hESCs (line H9)	WiCell	Cat# 16-W0060
hiPSCs (IMR90-4)	WiCell	Cat# 17-W0063
**Experimental Models: Organisms/Strains**		
Mouse: NOD-Prkdcem26Cd52/Gpt (NOD-SCID)	GemPharmatech	Cat# T001492;
**Software and Algorithms**		
Adobe Illustrator CS6	Adobe Systems	https://www.adobe.com/products/illustrator.html ; RRID: SCR_010279

Adobe Photoshop CS6	Adobe Systems	https://www.adobe.com ; RRID: SCR_014199
GraphPad Prism 7 software	GraphPad	https://www.graphpad.com ; RRID: SCR_002798
IBM SPSS Statistics 22 software	IBM SPSS Statistics	https://www.ibm.com/products/ spss-statistics RRID: SCR_019096
ImageJ	ImageJ	https://imagej.nih.gov/ij ; RRID: SCR_003070
Mini Analysis Program 6.0	Synaptosoft, Inc.	http://www.synaptosoft.com/MiniAnalysis ; RRID: SCR_002184
pClamp 10	Molecular Devices	http://mdc.custhelp.com ; RRID: SCR_011323
ZEN2009 Light Edition	Carl Zeiss	https://www.zeiss.com ; RRID: SCR_013672
**Other**		
borosilicate Glass	Sutter Instrument	Cat# BF150-86-10
borosilicate Glass Capillaries	World Precision Instruments	Cat# TW100-4

Supplementary Table 2. Summary table showing the replicates number of organoids and mice used for immunostaining, behavioral, and electrophysiological experiments

Experiment	Figure number	Group	Replicates number	Organoids origin
Assessment of infarct area	Supplementary Fig. 2a-b	PT-1	mice number, n=10	N/A
		PT-2		
Behavioral test	Fig. 5a-d	NIM + sham	mice number, n=9	N/A
		NIM + stroke	mice number, n=12	
		Organoids + stroke	mice number, n=14	hESCs (line H9)
	Fig. 5h-k	Sham treated with vehicle	mice number, n=9	N/A
		Stroke treated with vehicle	mice number, n=12	
		Organoids + stroke treated with vehicle	mice number, n=14	hiPSCs (IMR-90-4)
		Organoids + stroke treated with CNO	mice number, n=14	
	Supplementary Fig. 2c-f	Sham	mice number, n=10	N/A
		PT-1		N/A
		PT-2		N/A
	Supplementary Fig. 13a-d	NIM + sham treated with vehicle	mice number, n=13	N/A
		NIM + sham treated with CNO		N/A
		NIM + stroke treated with vehicle		N/A
		NIM + stroke treated with CNO		N/A
Immunostaining for organoids in vitro	Supplementary Fig. 3a-h	FOXG1$^+$/HO	Organoids, n=18	hESCs (line H9)
		Tuj-1$^+$/HO		
		PAX6$^+$/HO		
		TBR1$^+$/HO		
		CTIP2$^+$/HO		
		GAD67$^+$/HO		
		Ki67$^+$/HO		

		NeuN$^+$/HO		
	Supplementary Fig. 3k-n	FOXG1$^+$/HO	Organoids, *n*=10	hiPSCs (IMR-90-4)
		PAX6$^+$/HO		
		TBR1$^+$/HO		
		CTIP2$^+$/HO		
	Supplementary Fig. 5b	SOX2$^+$/HO	Organoids, *n*=18	hESCs (line H9)
		PAX6$^+$/HO		
		TBR2$^+$/HO		
Immunostaining for organoids in vivo	Fig. 2f-h	HN$^+$/HO	brain slices from 6 mice	hESCs (line H9)
		NeuN$^+$/HO		
		GFAP$^+$/HO		
		NeuN$^+$HN$^+$/NeuN$^+$		
		NeuN$^+$HN$^-$/NeuN$^+$		
		GFAP$^+$HN$^+$/GFAP$^+$		
		GFAP$^+$HN$^-$/GFAP$^+$		
	Fig. 5f	mCherry$^+$HN$^+$/HN$^+$		hiPSCs (IMR-90-4)
	Supplementary Fig. 5a	SOX2$^+$/HN$^+$		hESCs (line H9)
		PAX6$^+$/HN$^+$		
		TBR2$^+$/HN$^+$		
	Supplementary Fig. 6	SOX2$^+$/HN$^+$		
		PAX6$^+$/HN$^+$		
		TBR2$^+$/HN$^+$		
		TBR1$^+$/HN$^+$		
		FOXP2$^+$/ HN$^+$		
		CTIP2$^+$/ HN$^+$		
		SATB2$^+$/ HN$^+$		
		Ki67$^+$/HN$^+$		
		NeuN$^+$/HN$^+$		
		BRN2$^+$/GFP$^+$		
		CAMKII$^+$/ GFP$^+$		
		STEM123$^+$/ GFP$^+$		
		GAD67$^+$/GFP$^+$		
	Supplementary Fig. 7	TBR1$^+$/HN$^+$		
		CTIP2$^+$/ HN$^+$		
		SATB2$^+$/ HN$^+$		
		NeuN$^+$/HN$^+$		
		DCX$^+$/HN$^+$		

		Ki67$^+$/HN$^+$		
		GFAP$^+$/HN$^+$		
		OLIG2$^+$/HN$^+$		
		GABA$^+$/HN$^+$		
		OLIG2$^+$HN$^+$/OLIG2$^+$		
		OLIG2$^+$HN$^-$/OLIG2$^+$		
	Supplementary Fig. 10	mCherry$^+$HN$^+$ /mCherry$^+$		hiPSCs (IMR-90-4)
		mCherry$^+$HN$^-$ /mCherry$^+$		
Survival percent	Supplementary Fig. 12a	NIM + sham	mice number, *n*=10	N/A
		NIM + stroke	mice number, *n*=12	N/A
		Organoids + stroke	mice number, *n*=15	hESCs (line H9)
Body weight	Supplementary Fig. 12b	NIM + sham	mice number, *n*=9	N/A
		NIM + stroke	mice number, *n*=12	N/A
		Organoids + stroke	mice number, *n*=14	hESCs (line H9)
Electrophysiology	Fig. 2l	Number of APs evoked	*n* = 13 neurons from 5 mice	hESCs (line H9)
	Fig. 2m	sPSCs	n = 34 neurons from 7 mice	
	Fig. 4f	hChR2-mCherry mediated sPSCs recordings	*n* = 13 neurons from 5 mice, 6 recorded neurons were responsive to photo-stimulation.	
	Fig. 4g			
	Fig. 4i	LFP (contralateral motor cortex)	*n* = 7 slices from 3 animals	hESCs (H9-CAG-ChR2-EYFP)
	Fig. 4j	LFP (ipsilateral striatum)	*n* = 6 slices from 3 animals	
	Supplementary Fig. 11f	ChR2-EYFP mediated sPSCs recordings	*n* = 19 neurons from 6 mice, 6 neurons were responsive to photo-stimulation.	
	Supplementary Fig. 11g			
